# Nanomedicine for Gene Delivery and Drug Repurposing in the Treatment of Muscular Dystrophies

**DOI:** 10.3390/pharmaceutics13020278

**Published:** 2021-02-19

**Authors:** Ilaria Andreana, Mathieu Repellin, Flavia Carton, David Kryza, Stéphanie Briançon, Bénédicte Chazaud, Rémi Mounier, Silvia Arpicco, Manuela Malatesta, Barbara Stella, Giovanna Lollo

**Affiliations:** 1 Laboratoire d’Automatique, de Génie des Procédés et de Génie Pharmaceutique, Université Claude Bernard Lyon 1, CNRS UMR 5007, 43 bd 11 Novembre 1918, 69622 Villeurbanne, France; ilaria.andreana@unito.it (I.A.); mathieu.repellin@univr.it (M.R.); david.kryza@univ-lyon1.fr (D.K.); stephanie.briancon@univ-lyon1.fr (S.B.); 2 Department of Drug Science and Technology, University of Turin, Via P. Giuria 9, 10125 Torino, Italy; silvia.arpicco@unito.it; 3 Department of Neurosciences, Biomedicine and Movement Sciences, Anatomy and Histology Section, University of Verona, Strada Le Grazie 8, 37134 Verona, Italy; flavia.carton@uniupo.it (F.C.); manuela.malatesta@univr.it (M.M.); 4 Department of Health Sciences, University of Eastern Piedmont, Via Solaroli 17, 28100 Novara, Italy; 5 Hospices Civils de Lyon, 69437 Lyon, France; 6 Institut NeuroMyoGène, University of Lyon, INSERM U1217, CNRS UMR 5310, 8 Avenue Rockefeller, 69008 Lyon, France; benedicte.chazaud@inserm.fr (B.C.); remi.mounier@inserm.fr (R.M.)

**Keywords:** nanoparticles, Duchenne Muscular Dystrophy, myotonic dystrophy, antisense oligonucleotides, small molecules, CRISPR/Cas9

## Abstract

Muscular Dystrophies (MDs) are a group of rare inherited genetic muscular pathologies encompassing a variety of clinical phenotypes, gene mutations and mechanisms of disease. MDs undergo progressive skeletal muscle degeneration causing severe health problems that lead to poor life quality, disability and premature death. There are no available therapies to counteract the causes of these diseases and conventional treatments are administered only to mitigate symptoms. Recent understanding on the pathogenetic mechanisms allowed the development of novel therapeutic strategies based on gene therapy, genome editing CRISPR/Cas9 and drug repurposing approaches. Despite the therapeutic potential of these treatments, once the actives are administered, their instability, susceptibility to degradation and toxicity limit their applications. In this frame, the design of delivery strategies based on nanomedicines holds great promise for MD treatments. This review focuses on nanomedicine approaches able to encapsulate therapeutic agents such as small chemical molecules and oligonucleotides to target the most common MDs such as Duchenne Muscular Dystrophy and the Myotonic Dystrophies. The challenge related to in vitro and in vivo testing of nanosystems in appropriate animal models is also addressed. Finally, the most promising nanomedicine-based strategies are highlighted and a critical view in future developments of nanomedicine for neuromuscular diseases is provided.

## 1. Introduction

Muscular dystrophies (MDs) are a group of chronic inherited genetic diseases, with a worldwide estimated prevalence of 19.8–25.1 per 100,000 persons [1,2]. These multi-organ diseases mainly affect muscles, especially skeletal muscles, which undergo a progressive degeneration causing severe health problems that lead to poor life quality, loss of independence, disability and premature death [3,4]. Among the various types of MDs described so far, the most commons are the Duchenne Muscular Dystrophy (DMD) and Myotonic Dystrophies (DMs) [2,3,5].

Currently, no therapies are available to counteract the pathogenic causes of these diseases, and conventional treatments based on immunosuppressants such as corticosteroids or anti-inflammatory treatments are aimed to only mitigate symptoms [6,7,8,9,10]. Although corticosteroids are considered as the “gold standard” to preserve muscle strength in MDs, especially in DMD, their long-term administration is associated with serious adverse effects such as leg oedema, glaucoma, depression, hypertension, hyperglycaemia, osteoporosis, osteonecrosis and fractures [11,12,13]. Medical devices such as pacemaker, respiratory assistance or wheelchairs, rehabilitative therapy and as a last resort surgery are also widely used in the management of MDs patients [14,15,16]. Altogether, these approaches represent the current restricted therapies and methods approved to reduce pain and improve the quality of life. Recently, a variety of possible therapeutic strategies have been proposed such as repurposing US Food and Drug Administration (FDA)-approved molecules for the treatment of other diseases or developing novel gene therapy for exon-skipping strategy or genome editing [17,18,19,20,21,22]. However, due to their rapid clearance or associated adverse effects, further refinements are required to enter clinics.

Over the last several years, drug delivery nanosystems, referred to as nanomedicine, have been extensively explored for the development of more effective and safer treatments with main applications in cancers [23,24,25,26], central nervous system-related disorders [27,28,29] and immune diseases [30,31,32]. More recently, nanomedicine has also been investigated for the treatment of viral infections [33] such as the lately approved Moderna’s and Pfizer’s Covid-19 nanoparticle-based vaccines [34,35,36,37]. In cancer therapy, nanomedicine holds potential to improve current treatments by reducing side effects of chemotherapeutic agents. Moreover, combination approaches and immunomodulation strategies have been successfully developed to boost their performances [38,39,40]. Nevertheless, only 15 nanoparticle-based cancer therapies have received clinical approval and entered the market, such as the recent liposomal Onivyde^®^ and Vyxeos^®^ formulations [41,42].

Currently, novel nanomedicines are optimized for the treatment of skeletal muscle pathologies like MDs. However, multiple biological and pharmaceutical barriers challenge nanomedicine delivery to skeletal muscles. Biological barriers are embodied by the complex architecture of the skeletal muscle, which encompasses the skeletal muscle parenchyma itself, connective tissue, blood vessels and nerves. One of the main hurdles for delivery to skeletal muscles lies in the presence of the dense extracellular matrix (ECM), which accounts for 1 to 10% of the muscle mass [43,44,45]. Mostly made of fibrous-forming proteins (collagens, glycoproteins, proteoglycans and glycosaminoglycans) it hampers nanoparticles (NPs) penetration by retaining them in the ECM via electrostatic and mechanical interactions [46,47].

Pharmaceutical barriers encompass formulation and associated aspects related to scaling up of nanomedicine products. Recently, formulation techniques based on scalable processes have been developed to allow transposition of nanomedicine to industrial settings [48,49,50].

In addition to these barriers, an important requirement is that NPs have to be biocompatible to prevent additional muscle degeneration of severely injured skeletal muscles. For instance, sarcolemma membrane of DMD patients is severely affected and therefore more susceptible to damage by any treatment [51].

The present review aims at highlighting the major advances in the nanomedicine-based strategies for treating MDs. We reviewed the most recent approaches to treat MDs focused on oligonucleotides or antisense oligonucleotides, and small molecules and how nanocarriers have been designed to deliver them to muscle cells. In addition, the genome editing CRISPR/Cas9 system is also described. Finally, future perspectives for nanomedicine optimisation are presented.

## 2. Muscular Dystrophies Characterised by Gene Alteration

**Figure 1:**
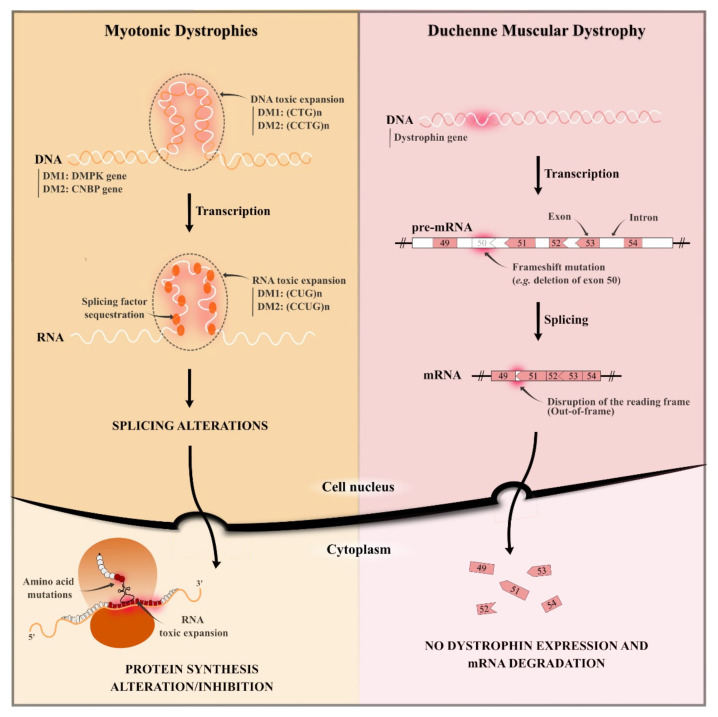
Schematic representation of the pathogenesis of both Myotonic Dystrophies and Duchenne Muscular Dystrophy. (DM1: Myotonic Dystrophy type 1; DM2: Myotonic Dystrophy type 2.)

Most common MDs are represented by the two well-known DMD and DMs [1,3,4]. These two types of dystrophies are caused by diverse gene mutations and lead to different molecular pathogenesis, as illustrated in Figure 1.

### 2.1. Duchenne Muscular Dystrophy

DMD is the most common form of MD characterised by progressive muscle degeneration and weakness, firstly affecting proximal muscles. This X-linked recessive rare disorder appears essentially in males with a worldwide prevalence of one in 5000 boys in early childhood and the clinical signs are not revealed at birth [52]. In most cases, the diagnosis is established around four years old, when the first symptoms start to appear. The disease progression is fast and the patients completely lose their motor functions around 10 years old. DMD patients develop also important cardiac and respiratory complications that generally manifest around 10 years old and are prevalent in most patients by 20 years old [53].

DMD is caused by mutations in the dystrophin gene (DMD gene) located on chromosome Xp21.2 that codes for the dystrophin protein through its 79 exons [54]. Dystrophin protein is essential for muscle homeostasis: it is localised on the plasma membrane of cardiac and skeletal muscles (sarcolemma), connecting the cytoskeleton to the ECM through the dystroglycan complex (DGC) and stabilising the muscle fibre during contraction. Lack of dystrophin induces severe muscle weakness, inflammation and wasting, described as cardinal signs of DMD. Diverse mutations on DMD gene have been reported worldwide [55]. Over the years, DMD gene has been characterised, identifying which mutations lead to a severe DMD phenotype [56,57]. Of DMD cases, 60–70% are caused by large deletions of one or more exons. Point mutations affect 15–30% of DMD patients, and there are smaller changes that do not involve an entire exon. Among them, nonsense mutations cause a premature stop in the gene which results in reduced dystrophin production or no production at all. Duplications affect only 10% of DMD patients and can occur throughout all 79 exons of the dystrophin gene. Understanding the nature of mutations improves the identification of therapeutic approaches able to rescue genetic mutations [58,59].

### 2.2. Myotonic Dystrophies (Type 1 and 2)

Myotonic dystrophies (DMs) are genetic disorders of autosomal dominance inheritance and represent the second most common form of MDs in adulthood [60,61]. These multisystemic diseases cause progressive dysfunctions of multiple organs and tissues (e.g., muscle tissues, skin, endocrine system, ocular system, central nervous system) among which skeletal muscle is the most severely affected tissue [5]. Muscle damages are characterised by progressive myopathy, muscle weakness, and progressive myotonia which is defined as a slow-down of muscle relaxation after a normal contraction. Most serious features concern the cardiopulmonary system and can lead to premature death, amounting to 70% of deaths [62].

Two distinct forms of DM caused by similar mutations are identified: i) DM1, also named Steinert disease (OMIM 160900), which is the most common and severe form; and ii) DM2, termed proximal myotonic myopathy (OMIM 602668). These DMs are caused by pathological expansions of small DNA sequences regarding two different genes [63]. DM1 is due to a (CTG)n expansion in the 3′ UTR region of the DMPK gene, while DM2 is caused by a (CCTG)n expansion in the first intron of the ZNF9/CNBP gene. Mutant (CTG)n and (CCTG)n expansions are highly unstable, leading to different repeat sizes constantly generated and increasing when transmitted from one generation to the next [64]. These mutant DNA expansions are transcribed into (CUG)n and (CCUG)n mutant RNA expansions for DM1 and DM2, respectively, aggregated in the nucleus in specific hairpin structures that are called nuclear foci [65].

The most accepted pathogenic hypothesis for DMs is an RNA-gain-of-function due to mutant RNA expansions that alter RNA-binding splicing regulators [66]. As illustrated in Figure 1, the main molecular hallmark of DMs is the sequestration of Muscleblind protein (MBNL), resulting in a local reduction of these protein levels [67]. It is responsible for several symptoms depending on the DM type and repeat size range, such as myotonia, muscle weakness, cardiac arrhythmia, diabetes, cataracts, male hypogonadism, cognitive disorders and hypersomnia [68]. Other splicing factors are mis-regulated in DMs, such as the up-regulation of CUGBP1 [63] and hnRNP H protein [69,70]. Understanding the molecular pathogenic mechanism helped the development and identification of diverse therapeutic approaches such as the reduction of toxic RNA levels [71,72], the prevention of the MBNL protein sequestration, or the inhibition of the signalling pathway that leads to CUGBP1 up-regulation [73,74,75].

## 3. Targets and How to Reach Them: DNA and RNA

**Figure 2:**
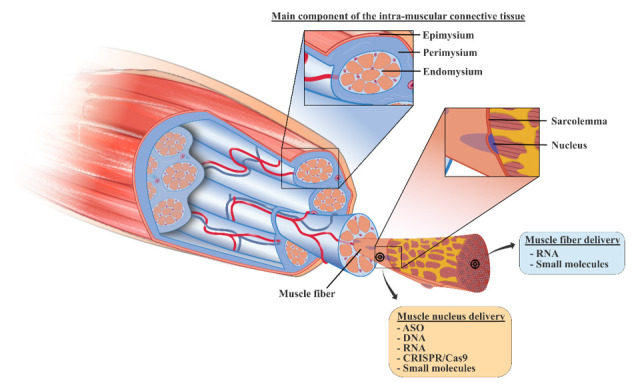
Organization of the skeletal muscle tissue and the target-tissue related to the class of experimental molecules (ASO, antisense oligonucleotide; CRISPR, clustered regularly interspaced short palindromic repeats).

MDs are genetic disorders caused by localised mutations of DNA. Such DNA mutations result in a lack of dystrophin protein in DMD and an alteration of the protein production in DMs (Figure 1) [57,76]. No curative therapies are available to treat the pathogenic causes, and the identification of new therapeutic approaches to target genetic mutations is urgently needed [10]. The most recent strategies to counteract MDs concern gene therapy and repurposing of drugs, as summarised in Table 1. In order to reach the target and achieve therapeutic effect, active agents must attain skeletal muscle tissue. A representation of their localisation is reported in Figure 2.

### 3.1. Gene Therapy and Genome Editing for MDs

Gene therapy aims at targeting gene mutations. Genetic strategy and genome editing offer the advantage of a durable and possibly curative approach. They aimed at improving patient’s quality of life by i) reducing the administration frequency, thus increasing their compliance to the treatment [97,98], and ii) providing a personalised therapy for the correction of genetic mutations [99].

Application of gene therapy in muscle pathologies is performed by exon-skipping [58] and CRISPR/Cas9 system [59] Exon-skipping is based on the use small of pieces of modified oligonucleotides, termed antisense oligonucleotides (ASOs), which recognise and bind specific sequences of mRNA [58,100]. ASOs exert their activity in the nucleus by: (i) blocking protein maturation and splicing alteration of pre-mRNA, (ii) degrading targeted RNA by RNase H-enzyme and reducing mRNA level [101]. Despite their promising features, drawbacks related to ASO’s instability, limited distribution to all diseased tissues, rapid body clearance and difficulties of cellular internalisation have been described [102]. To overcome these issues, chemical modifications on ASO chemical structure have been studied. Several ASO drugs were modified using the phosphorothioate backbone modification where one of the non-bridging oxygen atoms of the phosphodiester linkage was replaced with sulphur. This modification increased the half-life of ASOs [103] and reduced their degradation by exonucleases [104]. However, ASO-based therapies present potential toxicities such as the off-target RNA hybridisation and the possibility to alter protein expression [105]. In addition, they can be recognised by the immune system, inducing proinflammatory effects [106].

In DMD patients affected by an out-of-frame mutation of DMD gene, ASO therapy can restore the reading frame of mRNA leading to the expression of a partially functional dystrophin protein [107]. Eteplirsen was approved in 2016 by the FDA as the first antisense therapy for DMD. Eteplirsen is a 30-nucleotide phosphorodiamidate morpholino (PMO) ASO that resulted in an increased dystrophin production in all patients treated by weekly intravenous (I.V.) infusion for at least 24 weeks [77]. Drisapersen, a 2′-O-methyl-phosphorothioate ASO, is another exon-skipping therapy for DMD in clinical development. Phase 3 study (DMD114044; NCT01254019) evaluated the efficacy of drisapersen after subcutaneous (S.C.) injection. The six-minute walk distance (6MWD) was the primary endpoint considered, and no differences were reported between placebo and treated patients. Data analysis considering secondary endpoints such as the North Star Ambulatory Assessment (NSAA), 4-stair climb ascent velocity and 10-metre walk/run velocity, showed lack of statistical significance due to the greater data variability and subgroup heterogeneity. However, statistically significant results were obtained in the young patients treated in the early stage of the pathology [78]. Further studies were focused on the preclinical optimisation of drisapersen, testing its efficacy on young and older DMD mouse model, *mdx*. This mouse model is characterised by an induced nonsense point mutation in the dystrophin gene, which leads to a loss of functional protein expression [108]. Preclinical investigation showed a comparable efficiency of exon-skipping mechanism in young and older mice, but functional and physical improvement was only reported for treated young mice, meaning that the stage of pathology is relevant for treatment efficacy. In vivo studies highlighted that an early intervention with drisapersen led to functional benefits despite the low level of dystrophin restoration. In addition, the application of drisapersen was also limited by the reported side effects at the injection site and mostly proteinuria, which increased α1-microglobulin levels [79]. Although ASO therapy and exon-skipping reached important outcomes, their applicability is limited to patients affected by out-of-frame mutation and requires continuous administrations throughout the lifetime of the patients [109,110].

ASO therapy is also reported for the treatment of DM where it targets and neutralises toxic CUG/CCUG^exp^ RNAs which sequestrate splicing factors [111]. Although there is no approved ASO therapy for DM1, in vitro and in vivo results are promising [112,113,114]. It has been reported that ASOs containing 2′- 4′- constrained ethyl (cEt) modifications can be employed to target DMPK genes and substantially reduce CUG^exp^ RNA nuclear foci in patient-derived DM1 myoblasts [80]. Klein et al. developed an arginine rich Pip6a cell-penetrating peptide-conjugated PMO ASO to overcome the poor distribution of ASOs in skeletal muscle. Pipa6-conjugated ASO directed against CUG^exp^ allowed an effective concentration of ASOs in muscle fibres recovering MBNL1-dependent splicing defects [81].

In contrast to ASO therapy, genome editing induces a permanent correction of gene mutation [115]. Initially, engineered zinc finger nucleases (ZFNs) and transcription activator-like effector (TALENs) have been used to permanently remove splicing sequences in DMD gene and to restore dystrophin expression [116,117]. Recently, Clustered Regularly Interspaced Short Palindromic Repeats (CRISPR) in association with specific DNA endonuclease protein called Cas9, targeting DNA sequencing, has been investigated to restore the genetic mutation [118,119,120]. Cas9 nuclease requires a single guide RNA (sgRNA) to form a complex with DNA by recognition with a defined 20 bp DNA sequence, known as protospacer. The protospacer sequence is immediately followed by a short sequence called protospacer-adjacent motif (PAM), needed by Cas9 for DNA cleavage and for genome editing to start [121].

Promising results of CRISPR/Cas9 in MD treatment were reported. Dystrophin recovery by CRISPR/Cas9 system was observed in a new DMD mouse model characterised by a lacking exon 44 of the dystrophin gene, one of the hotspot regions for DMD gene mutation. In this study, Cas9 and sgRNA, the main gene editing components, were encoded by adeno-associated viruses serotype 9 (AAVs). The ratio between AAVs encoding for Cas9 and for sgRNA had an important effect on gene correction. Higher level of sgRNA ensured higher Cas9 activity, which increased dystrophin restoration due to long-lasting presence of sgRNA that allows continuous editing in myofibers. Combination of optimized sgRNA and AAV vectors for delivery increased long-term correction of dystrophin mutation in mice [82]. AAV9 have been already associated to CRISPR/Cas9 system for dystrophin recovery in a deltaE50-MD canine model of DMD, which leads to loss of exon 50. An sgRNA was optimised to target a region adjacent to the exon 51 splice acceptor site, and it resulted in high frequency of reframing events. Dystrophin protein expression was restored to 60% by intramuscular (I.M.) injection of AAV9-Cas9 and AAVs-sgRNA-51 [83]. Besides, CRISPR/Cas9 ability to target repeated DNA sequences also provides a possible strategy in DM therapy [122]. Dastidar et al. evaluated CRISPR/Cas9 activity, delivered through viral vector to excise CTG repeats in DM1 patient-derived cells, leading to the normalisation of DMPK gene expression and the degradation of toxic RNAs. The study demonstrated the potential application of CRISPR/Cas9 excision in trinucleotides repeat expansion up to 1200 repeats [88].

Genome editing by CRISPR/Cas9 strategy requires an efficient delivery system. AAV are used most often and have a low cargo capacity as compared with other viral vectors, requiring a high AAV dose to deliver sgRNA for gene reprogramming in vivo [21], increasing the risk of immunogenicity [123]. Traditional genome editing based on CRISPR/Cas9 technologies introduces double-stranded (ds) DNA breaks at a target locus as the first step to gene correction. However, the potential applications of Cas9 nucleases are limited in part by their reliance on DNA breaks, which could cause deletions, insertions or chromosomal rearrangements. Due to recent advances, CRISPR/Cas9-associated base editing (BE) approaches have emerged. These strategies do not require a dsDNA backbone but mediate the direct conversion of base pairs, advancing the treatment of genetic disorders associated with single nucleotide mutations [124,125]. Cytosine and adenine base-editors are the most used tools to exert mutation transition (Cytosine (C) > Thymine (T) and Adenine (A) > Guanine (G)), guided by a new CRISPR/Cas9 system which targets the non-edited DNA strand [126]. Ryu et al. demonstrated the application of BE in DMD treatment, using a dual trans-splicing AAV to deliver adenine base editors (ABE). ABE treatments achieve a precise A-to-G base mutation, restoring dystrophin expression in 17% of myofibers, following I.M. into tibialis anterior in *mdx* mice [127]. Moreover, due to increased interest in BE, non-viral vectors are under investigation to replace viral constructs as delivery systems [21]. Jiang et al. demonstrated a successful delivery of ABE using lipid nanoparticles in Tyrosinemia I mice, correcting the gene mutation and showing the promise of the BE approach [128]. Despite the far-reaching capabilities of the BE strategy, a major limitation of this technique has been the ability to generate precise edits beyond the allowed transition mutations. Anzalone et al. described a new genome editing strategy called prime editing. This search-and-replace technology directly writes new genetic information into targeted DNA using a catalytically impaired Cas fused to an engineered reverse transcriptase enzyme, and a prime editing guide RNA (pegRNA) able to recognise and bind the target site [129]. This technology with its simplicity and precision holds great promise for the correction of point mutation in human genetic disorders.

### 3.2. Drug Repurposing

Another current approach in MD treatment is drug repurposing, a strategy for identifying new applications for approved or investigational drugs that are outside the scope of the original medical indication. Drug repurposing has promising expectations regarding efficacy, safety, cost and translation to the clinical setting. This is because repurposed drugs have been already studied in preclinical models and humans for safety assessments. Moreover, in many cases the formulation aspects are already developed [130].

In order to select the right candidate in a repurposing strategy, a systematic approach that combines computational techniques and experimental studies is required. Computational approaches are based on data-analysis (gene expression, chemical structure, genotype or proteomic data, or electronic health records (EHRs)), to validate the repurposing hypothesis. Experimental approaches are also required to identify target interactions and efficacy in appropriate models [131]. Table 1 lists the most common drugs used for repurposing strategies in DMD and DM.

About 10% of DMD patients present a nonsense mutation, which induces a premature stop codon in dystrophin mRNA leading to non-functional protein [132,133,134]. Some compounds are able to bind the stop codon, forcing the translational machinery to incorporate amino acids into the assembling protein, overcoming the stop signal and obtaining a functional protein [87]. Restoration of dystrophin protein was studied using gentamicin, an aminoglycoside antibiotic made of a mixture of major and minor aminoglycoside components [88]. Barton-Davis et al. demonstrated the possibility of treating DMD nonsense mutation using gentamicin. The drug was administered by S.C. injection to *mdx* mice at different dosages to identify the optimal dose to restore the full-length dystrophin [87,89]. To determine the efficacy on the suppression of premature stop codon in *mdx* mice, evaluation of dystrophin protection against contraction-induced damage was examined. The number of damaged fibres was reduced in treated *mdx* mice, as compared with wild type mice. Prolonged use of gentamicin implicates nephrotoxicity, limiting the long-term administration required for genetic diseases [135]. To address toxicity issues, new aminoglycosides and non-aminoglycosides were explored. Friesen et al. demonstrated the efficacy and greater read through-safety window than other compounds, of a minor gentamicin component called gentamicin X2, which shows a lower toxicity than gentamicin [136]. From the evaluation of neuromast toxicity (cytotoxic concentration, CC50), as a substitute for ototoxicity, CC50 of gentamicin X2 was significantly reduced compared to gentamicin. Minor component X2 has great potential for clinical utility in treating genetic diseases caused by nonsense mutations. Among non-aminoglycoside compounds, ataluren is a novel, orally administered, synthetic molecule that suppresses nonsense mutation in a way similar to aminoglycosides and increases dystrophin production [90]. Data from a phase III trial did not show improvement in 6MWD of treated patients, but less physical deterioration was demonstrated for patients receiving ataluren than for those receiving placebo. Results reported in this trial confirmed the clinical benefit of ataluren in terms of preservation of muscle function in DMD patients [91]. Further studies should evaluate the long-term benefits the drug.

In DM, the toxic foci made of CUG/CCUG^exp^ RNA aggregates are able to sequestrate MBNL1 splicing factor, thus altering the protein expression [61]. As an example, pentamidine, furamidine and erythromycin can inhibit the sequestration of splicing factors such as MBNL1 by CUG^exp^ RNA sequence. Docking analyses are useful to predict the binding conformation of small molecules to appropriate binding sites. They have been reported for rational screens of molecules that might selectively bind CUG structures and consequently improve biological activity in DM1 models [137].

Recently, pentamidine, a diamine compound that FDA approved for the treatment of trypanosomiasis and leishmaniasis infections [138], has been proposed for MD treatment and its efficacy was evaluated in vitro and in vivo. Pentamidine treatment reduced CUG^exp^ RNA level and rescued mis-splicing events in HeLa DM1 transfected cells, which expressed 960 interrupted CUG repeats. The number of nuclear foci was reduced by 21% [139]. The efficacy of pentamidine treatment was evaluated on DM1 mouse model HSA^LR^ (human skeletal actin long repeat length) that expresses ~250 CUG repeats into the final exon of human skeletal actin [18]. Pentamidine was administered by intraperitoneal (I.P.) injection and the correction of chloride-1 and Serca1 mRNA mis-splicing was essayed. The treatment partially reversed mis-splicing events. In vivo studies highlighted a narrow dosage window for pentamidine which cannot be used over 30 mg/kg twice a day. To overcome dosage toxicity, chemical modifications were performed to enhance the specificity to CUG^exp^ RNA and to reduce the dosage. Based on the chemical backbone of pentamidine, other drugs showing chemical similarities were selected. Furamidine is a diamine compound that rescues the mis-splicing events in vitro and in vivo models [92]. In HSA^LR^ mice, furamidine increased MNBL1 functional expression by inhibiting transcription of CTG^exp^ DNA and by disrupting the MNBL-CUG^exp^ complex [93]. Compared to pentamidine, furamidine presented the lowest number of off-target gene expression changes [94]. In combination with erythromycin, furamidine enhanced the effects on MNBL-CUG^exp^ complex disruption, reducing nuclear foci presence in patient-derived DM1 cells, without specific toxic effects [95]. Considering the reduction of MBNL1 in DM1 affected cells, small molecules improve the pathological condition by overexpression of splicing factors. For example, ISOX and vorinostat were tested in normal and DM1 fibroblasts, increasing MBNL1 expression and revealing positive effects on DM1 models [96].

Globally, repurposing of small molecules for the treatment of DM requires extensive investigation concerning their off-label use and the need of novel approaches to define their therapeutic potential for a different disease.

## 4. New Treatments based on Nanocarriers as Alternative Strategies to Facilitate Skeletal Muscle Targeting

Over the last years, the application of nanomedicine as a promising innovative approach to treat different pathologies such as MDs has been investigated. The architectural and structural complexities of skeletal muscles challenge nanomedicine delivery, especially due to the important presence of ECM [45,140]. To restrict interactions with ECM, administration of NPs by I.V. appears as a potential strategy for targeting skeletal muscle. The dense blood capillary network of skeletal muscles increases NPs access to muscle fibres [141,142]. However, once in the blood circulation, NPs can be rapidly cleared through the mononuclear phagocyte system via opsonisation or complexation with plasma proteins [143,144,145,146]. Physical and chemical instability [147,148], immunogenicity [149,150] or premature degradation [151] are other limiting factors that might interfere with NPs delivery.

**Figure 3:**
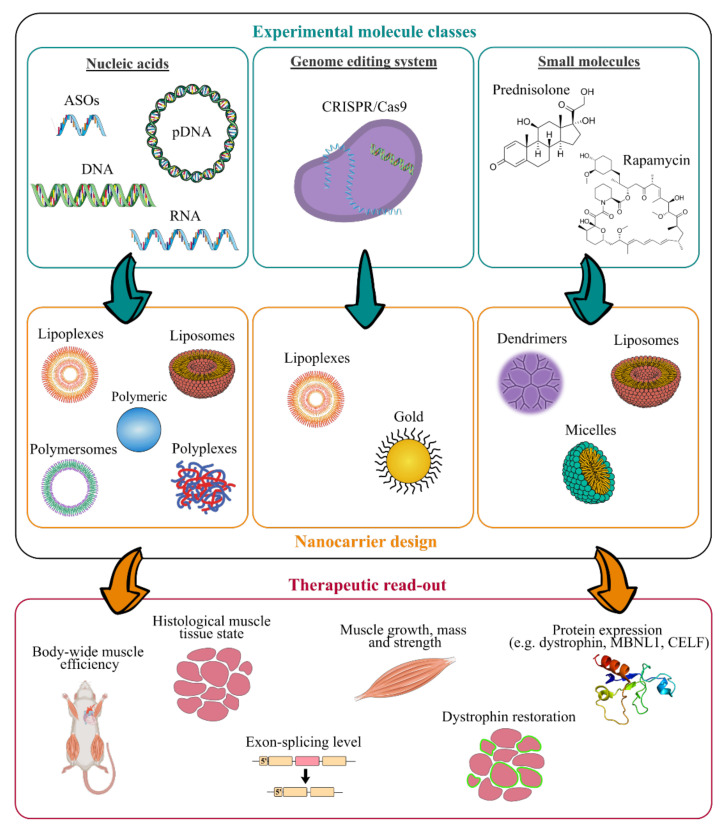
Work flow for the design of innovative nanomedicine and therapeutic readout. (ASOs, antisense oligonucleotides; CRISPR, clustered regularly interspaced short palindromic repeats).

In addition, long-term administration is required to cure chronic disorders such as MDs, which makes biocompatibility and biodegradability of the nanosystems important requirements [152]. NPs should persist long enough to reverse muscle damages without involving any additional muscle degeneration, before undergoing gradual degradation [51,153]. Therefore, their design has to be optimised to associate or encapsulate active compounds and to deliver them to skeletal muscles. As illustrated in Figure 3, various NPs structures have been described [154]. RNA- and DNA-based nanocarriers are obtained via electrostatic and hydrophobic-hydrophobic interactions with polymers or lipids [155,156,157]. In the case of delivery of the small molecules, their chemical properties, such as their molecular size, structure and n-octanol-water partition coefficient have an impact on the selection criteria for nanocarrier strategy [158,159]. Interestingly, synthetic nanocarriers interacting by electrostatic and hydrophobic-hydrophobic interactions have been demonstrated to deliver complex CRISPR/Cas9 systems under various forms such as DNA, mRNA or ribonucleoproteins [160,161,162].

As illustrated in Figure 3, many experimental molecules and macromolecules have been selected as candidates for MD therapies, and a wide range of nanocarriers has allowed their delivery to skeletal muscles, promoting in most of the cases their therapeutic potential.

The present section aims at highlighting nanosystems used for DMD and DM applications that reached preclinical studies. An overview of the various described nanosystems is reported in Table 2.

### 4.1. Antisense Oligonucleotides

ASO-based therapy is a powerful tool for inducing post-transcriptional modifications and thereby regulating target genes. There are several classes of ASOs for therapeutic purposes which differ from their phosphate backbone and ribose sugar group modifications [185]. Most ASOs used for DMD and DM are 2′O-methyl (2′O-Me), phosphorothioate (PS) ASO and PMO oligomer modified ASOs. Depending on their chemistry, different strategies can be obtained to modulate gene expression.

The phosphodiester and phosphorothioate internucleotide linkages confer a highly negative charge to PS or 2′O-Me ASOs. Hence, the most common approach to incorporate such ASOs into NPs remains to form stable complexes with cationic polymers or lipids. Polyplexes have been currently used for this purpose [186]. Poly(ethylene imine) (PEI) was one of the first cationic polymers explored for gene therapy because of its efficient binding association to nucleic acids and good transfection efficiency [187]. Nonetheless, the high positive surface potential results in significant toxicity, especially for in vivo skeletal muscle delivery, due to interactions with many biological components. To reduce the surface potential, poly(ethylene glycol) (PEG) has been added to the formulation [188,189]. Lutz’s group developed PEI-PEG NPs obtained by the complexation of 2′O-Me ASOs with a cationic copolymer of PEI and PEG, to restore dystrophin expression in *mdx* mice [163,190,191]. PEI was conjugated to nonionic linear PEG to provide NPs with a steric shield, greatly improving their biocompatibility. PEI-PEG-ASOs were shown to locally improve the levels of dystrophin expression up to 20% of normal dystrophin expression in WT animals after I.M. injection without eliciting toxicity. However, cationic PEI-PEG showed poor muscle distribution, evidenced by large untransfected areas in muscles, due to the nanoparticle entrapment into the ECM by non-specific binding [163].

In line with Lutz’s group, Sirsi et al. proposed a strategy to shield the positive charges of PEI-PEG-ASO polyplexes by encapsulating them into PLGA nanospheres [164]. In vitro release studies in physiological medium showed a low PEI-PEG-ASO release from PLGA nanospheres. Such slow release was correlated to the Mw of PLGA; high PLGA Mw (72 kDa) impaired complex release, while lower PLGA Mw (17 kDa) reached 66.5% release over 26 days. This different release span could be ascribed to the kinetic rate of hydrolysis of PLGA. However, in vivo studies in *mdx* mouse following I.M. injection of free or PLGA (17 kDa) encapsulated PEI-PEG-ASOs showed that dystrophin expression was not improved, probably related to an incomplete ASO release of PEI-PEG-ASOs from PLGA. Despite this lack of dystrophin expression, polyplexes encapsulation through PLGA nanospheres appears to be an efficient sustained delivery strategy of interest for treating chronic diseases.

Wang et al. designed PEI conjugated with Pluronic^®^ polycarbamates (PCM) to deliver 2′O-Me ASOs in *mdx* mice [166]. After I.M. administration, PEI-PCM NPs showed a local increased number of dystrophin-positive fibres up to three-eight fold, superior to ASO alone or unmodified ASO-PEI NPs. The addition of the carbamate hydrophobic groups contributed to ASO complexation to the NPs and enhanced transfection efficiency. 2′O-Me ASOs were also complexed using poly(ester-amine) (PEA), a constructed polymer obtained from PEI conjugated with Pluronic^®^. This polyplex demonstrated an efficacy similar to that of PCM with a number of dystrophin-positive fibres up to 3–10 fold higher than ASO alone [171].

Cationic polymethylmethacrylate (PMMA) NPs are other promising systems intended for nucleic acids delivery [192]. Rimessi et al. proved that cationic PMMA (named T1 NPs) complexed with 2′O-Me PS ASOs, administered by I.P. injection, restored dystrophin expression in body-wide striated muscles of *mdx* mice [168]. Dystrophin appeared expressed at moderate levels on the membrane of myofibers of the diaphragm, gastrocnemius and quadriceps and was restored at lower levels on the membrane of cardiomyocytes, showing the wide muscle distribution of T1 NPs. However, PMMA NPs are slowly degradable and might form small aggregates at high concentration in blood circulation, limiting their clinical use [192]. Based on these results, Ferlini et al. designed PMMA/N-isopropil-acrylamide+ (NIPAM) NPs (ZM2 NPs) to improve the potential of PMMA-based NPs [169]. PMMA core was shielded with NIPAM cationic copolymer and used to bind and convey 2′O-Me PS ASOs to *mdx* mice following I.P. administration. ZM2-ASOs NPs induced efficient and widespread dystrophin restoration at low dose of ASOs in both striated and smooth muscles, with a dystrophin expression up to 40% of muscle fibres and an exon-skipping level up to 20% after seven days. Moreover, these nanocarriers showed long-term residual efficacy over 90 days, demonstrating their potential as gene delivery systems for ASO delivery [170].

Other non-ribose modified ASOs less frequently used for MDs applications are PMO. These ASO modifications are aimed at increasing their nuclease resistance and mRNA binding efficacy, but provide poor cellular uptake and rapid blood clearance related to the uncharged nature conferred by the morpholino rings [193,194]. In contrast to negatively charged ASOs, the use of cationic entities as a delivery vehicle is not suitable with this well-established ASO delivery strategy. For efficient delivery of these neutral oligonucleotide analogues, lipophilic interactions through PMO and hydrophobic carriers are privileged [195]. Kim et al. showed, as proof of concept, the potential of non-ionic PEG-polycaprolactone and PEG-(polylactic acid) polymersomes for the I.M. administration of PMO for DMD application into *mdx* mice [167]. The polymersomes successfully enhanced dystrophin expression in the entire muscle length increasing three-fold the number of dystrophin-positive fibres as compared with free-PMO. Furthermore, these degradable carriers were biocompatible upon injection in the muscle, showing long-circulating properties with an improvement of dystrophin expression over three weeks after a single injection.

Wang et al. proved previously described PCM NPs for PMO delivery in *mdx* mice to be a strategy for DMD therapy [165]. Hydrophobic region of the carbamate groups enabled the formation of ASO-PCM carriers. These NPs dramatically improved dystrophin expression in muscles after I.M. injections with up to 57% of dystrophin-positive fibres, four-fold superior to ASO alone. After I.V. administration, up to 15% of myofibers were dystrophin-positive, resulting in a transfection efficiency 3-fold superior to ASO alone. In heart muscle, PCMs demonstrated an improvement of dystrophin-positive cardiomyocytes up to 5-fold superior, nevertheless leading to only 5% of dystrophin-positive cells. Hydrophobic PCM NPs showed high toxicity, whereas more hydrophilic PCM NPs were found ineffective to deliver PMO, emphasising the importance of a polymer’s hydrophobic and hydrophilic balance to improve charge-neutral PMO delivery.

Amphiphilic PEA NPs previously described for 2′-OMe ASO complexation, also proved to be efficient in delivering PMOs for DMD application in *mdx* mice [171]. PEA-PMOs induced three-fold more dystrophin positive myofibers than PMOs alone after both I.M. and I.V. injection, with the widespread presence of dystrophin-positive fibres in diaphragm, biceps and heart after systemic delivery.

Other works demonstrated that bubble liposomes combined with ultrasound exposure are an effective tool to enhance the delivery of PMOs in both HSA^LR^ and *mdx* mice for DM and DMD applications, respectively. The interesting transfection efficiency properties of these liposomes containing ultrasound imaging gas rely on their ability to cavitate under ultrasound exposure. This combination produces transient pores in cell membranes, enabling the direct entry of the therapeutic compounds into the cytoplasm without involvement of the endosomal pathway [196,197]. Koebis et al., demonstrated that bubble liposomes-PMO I.M. delivered in HSA^LR^ mice locally improved the alternative splicing of the chloride channel 1 (Clcn1) gene, downregulating the high Clcn1 protein level in DM muscles and thereby enhancing one of the multiple DM features [178]. To our knowledge, this is the only study on ASO delivery using nanomedicine for DM applications that has been tested in vivo. Negishi et al. showed the potency of identical bubble liposomes to deliver PMO for DMD application [177]. This combination of bubble liposomes and PMOs, followed by ultrasound exposure, locally restored dystrophin expression in *mdx* mice muscles with an exon 23-skipping improvement to less than two times and a number of dystrophin positive fibres ~five-fold superior as compared with PMO alone. Thus, the co-administration of bubble liposomes combined with ultrasound exposure may provide an effective non-invasive method for PMO therapy.

Overall, to effectively deliver ASOs in skeletal muscle, nanomedicine is a fundamental tool to ensure protection from degradation while improving both tissue and intracellular uptake. The equilibrium of the degree of hydrophobicity, Mw and charge potential is the key to ensure an optimum compromise between stable complex formation, efficiency and tissue compatibility. With regard to all studies presented above, nanomedicine has achieved encouraging improvement of ASO’s efficiency with special attention to skeletal muscles. Furthermore, systemic administration has showed promising results on the body-wide carrier distribution into skeletal, smooth and also cardiac muscles.

### 4.2. Oligonucleotides

RNA and DNA-based therapeutics are efficient and versatile strategies to regulate gene expression, making this class of drugs attractive for a variety of applications. Similar to negatively charged ASOs, strategies for oligonucleotides delivery are mainly based on electrostatic interactions [198,199,200,201].

Kinouchi et al. used atelocollagen (ATCOL) to condense a siRNA downregulating myostatin, a negative regulator of skeletal muscle growth [175]. ATCOL is a highly purified collagen chosen for its biocompatibility with skeletal muscle, as collagen occurs naturally as one of its principal components [43]. Moreover, it has been reported that ATCOL displays low in vivo immunogenicity and toxicity [202,203]. ATCOL-siRNA NPs markedly decreased the protein levels of myostatin and thereby increased muscle mass within two weeks after a single I.M. injection in *mdx* mice. The authors also demonstrated the potential of systemic ATCOL-siRNA delivery to repress myostatin expression, inducing muscle hypertrophy in normal mice.

Poly(2-aminoethyl propylene phosphate) (PPE-EA) polymer has also demonstrated good potential for pDNA delivery to the muscle tissue in healthy Balb/c mice [204]. The high PPE-EA molecular weight ensures suitable hydrolytic stability of the PPE-EA/pDNA complexes. Furthermore, the cleavage of the PPE-EA phosphoester bond in physiological conditions gives this polymer interesting biodegradability properties. In vivo gene transfer efficiency was evaluated using LacZ, coding for β-galactosidase, as a model gene. After I.M. injection, PPE-EA/pDNA demonstrated a significantly higher and delayed β-galactosidase expression in muscles, up to 17-fold superior to DNA alone, highlighting the great potential of PPE-EA polymer as muscle gene delivery [174].

Itaka et al. designed PEG-poly(L-lysine) (PEG-PLys)/pDNA NPs-mediated gene delivery systems for skeletal muscle in order to find novel strategies to inhibit tumour growth through the extensive capillary network wrapped around the muscle fibres [173]. The authors designed PEG-PLys to reconcile DNA binding affinity within cationic PLys core, and tolerance under physiologic conditions through the electrically neutral shell of PEG [205]. The transgene expression efficacy in the skeletal muscle of healthy Balb/c mice was evaluated using pDNA encoding luciferase and GFP, as a model gene. After injection into the blood stream of murine muscle limbs, PEG-PLys/pDNA induced a luciferase expression up to 10-fold higher than pDNA alone in the injected muscle over 25 days. Moreover, PEG-PLys/pDNA promoted an increased number of fluorescent-positive muscle fibres compared to pDNA alone, a better time-dependent profile of transgene expression without any overt signs of toxicity. Thus, PEG-PLys NPs provide prolonged transgene expression in skeletal muscle, which is promising for the field of muscle pathologies.

Hyperbranched poly(ester amine)s (demonstrated an efficient PEAs) obtained from chemical modifications of PEI also demonstrated interesting properties as pDNA delivery carriers in C2C12 murine muscle cells and *mdx* mice [172]. The authors cross-linked low-molecular-weight PEI polymers to raise a dispersed positive charge density into the core and enhanced gene transfection efficiency while reducing PEI associated cytotoxicity. These biodegradable NPs effectively condensed a pDNA coding for GFP and showed a low in vitro cytotoxicity on muscle cells. PEAs/pDNA complexes in vitro transfection with up to 87% of fluorescent-positive C2C12 muscle cells, suggesting a transfection efficiency 2–3-fold higher than that of cells transfected with PEI/pDNA. Finally, these nanosystems also proved to have good potential for muscle gene delivery, showing a substantial increase in fluorescent-positive muscle fibres after I.M. administration.

In a way similar to ASOs, the main strategies adopted to deliver oligonucleotides into skeletal muscle rely on the use of materials ensuring electrostatic and hydrophobic interactions. Finding a compromise between hydrophobic and charge degree is crucial in gene delivery to ensure carriers stability and great transfection efficiency while sustaining high tissue integrity. Although these studies have been presented as promising proof-of-concept for skeletal muscle delivery, further investigations using oligonucleotides designed for treating MDs could warrant the use of such nanocarriers to critically cure affected muscles.

### 4.3. Small Molecules

Drug repurposing is an effective strategy to reuse existing licensed drugs for novel medical indication while reducing development time, costs and minimising risk of failure [112,206]. In addition, nanomedicine has been used to enhance therapeutic potential of drugs with restricted pharmacological profile and encompass toxicity limitations and poor availability [207,208,209,210].

One of the first small molecules encapsulated for MD applications was gentamicin, to enhance its poor delivery profile to the muscle tissue and to decrease its toxicity [120,181]. In order to overcome these drawbacks, Yukihara et al. encapsulated gentamicin into liposomes made of phosphatidylcholine or phosphocholine and PEG. Efficient accumulation of these nanosystems in the cytoplasm and cytoplasmic membranes of myofibers after I.P. injection was observed. Gentamicin-loaded liposomes increased the percentage of dystrophin positive myofibers up to 7.7% compared to 2.4% for gentamicin alone and were able to suppress the drug related ototoxicity and nephrotoxicity. However, despite the positive features of liposomes, which led to an enhanced pharmacological profile of gentamicin, this small molecule is candidate for only nonsense DMD mutations, thus restricting its clinical potential only to a narrow population of DMD patients [211].

Regulating downregulated myogenic recovery factors such as Myogenic differentiation 1 (MyoD) appears to be a good strategy to restore muscle differentiation and regeneration [212]. For this purpose, Afzal et al. designed nanolipodendrosomes loaded with synthetised MyoD and glatiramer acetate (GA), a synthetic drug that increases the level of anti-inflammatory cytokines [179]. The results demonstrated that these loaded nanosystems significantly improved muscle mass of lower-limbs in healthy SW-1 mice after I.M. injection, while no improvement was observed in the other muscles. Authors suggested that further research would warrant the use of nanolipodendrosomes loaded with these two candidate drugs to ameliorate muscle regeneration.

Bibee et al. developed lipid NPs of perfluorocarbon (PFC) to deliver rapamycin [182], an immunosuppressant and anti-inflammatory agent that proved to restore defective autophagy mechanism in *mdx* mice, associated with many side effects [213]. The main advantage of PFC NPs is their exceedingly good stability in blood, as they were originally developed as a blood substitute [214,215]. After systemic injection, nanocarriers were able to rescue a correct autophagy flux in *mdx* mice, thus improving both skeletal muscle strength and cardiac contractile performance. On the other hand, no equivalent improvement was achieved with conventional oral rapamycin administration delivered at a concentration even 10-fold superior, corresponding to the pharmacological doses. Furthermore, these muscle performance improvements were observed in young or adult wild-type mice as well as in aged mice, demonstrating the broad efficiency of this therapy. Clinically, potential deleterious consequences of rapamycin could be mitigated by the lower required dose administration of rapamycin once loaded into PFC NPs.

Lowering the severe side effects associated with chronic glucocorticosteroid administration has been investigated by Turjeman et al., encapsulating methylprednisolone hemisuccinate (MPS) into PEGylated nanoliposomes (NSSL) [180]. These smaller liposomes (80 nm) benefit from the inflamed tissues’ unique vascular abnormality, achieving muscle passive targeting and accumulation into the inflamed tissue [216,217]. NSSL/MPS were mostly internalised into the diaphragm of young *mdx* mice after I.V. injection, due to the multiple tissue damages occurring in this organ at this stage, resulting in NPs leakage from capillaries. A significant decrease of TGF-β1 protein level was observed in serum, as a result of protective effects against inflammation. NSSL/MPS significantly ameliorated osteoporosis in elderly *mdx* mice, whereas MPS alone further increased bone catabolic effects, increasing DMD phenotype. Finally, differences in NSSL/MPS treatment doses showed diverse muscle benefits: lower doses demonstrating advantages in terms of muscle strength, whereas higher doses showing benefits in terms of mobility.

Márquez-Miranda et al. developed hydroxyl-terminated poly(amidoamine) (PAMAM-OH) dendrimer as a carrier for angiotensin (1–7), an anti-atrophic bioactive heptapeptide highly beneficial in the treatment of skeletal muscle pathologies [176]. PAMAM-OH dendrimers were used to increase angiotensin (1–7) short half-life, hydrolytic stability and poor systemic distribution. To observe the anti-atrophic effect of angiotensin (1–7), the authors unilaterally immobilised lower hind limbs of normal mice for 14 days. Loaded nanosystems demonstrated the ability to restore muscle strength and recover fibres diameter of immobilised limbs to levels similar to non-immobilised limbs after I.P. administration, whereas angiotensin (1–7) alone did not induce any recovery. Moreover, bone micro-architectural structure in elderly *mdx* mice was less damaged over 58 weeks of NSSL-MPS administration compared to mice treated with MPS. Altogether, these findings highlight the potency of PAMAM-OH/angiotensin (1–7) nanocarriers as an efficient general treatment of dystrophies for mitigating muscle atrophy.

Many therapeutic approaches have been explored through the screening of small molecules to reverse pathological consequences of MDs. Despite several advantages including lower costs, ease of therapy management and faster development process, small molecules are intended only to target downstream effects at the splicing or protein level and not to correct mutations at the DNA or RNA levels. In the studies presented above, nanomedicine has allowed for an increase in the therapeutic impact of experimental small molecules or well-known effective molecules, while significantly decreasing potential deleterious side effects.

### 4.4. CRISPR/Cas9 System

CRISPR/Cas9 system is a recent and powerful genome editing tool of great interest to treat genetic disorders such as DMD and DM [100,218]. The delivery of Cas9/sgRNA ribonucleoprotein complexes via non-viral delivery systems has been investigated to boost its clinical applications [219,220]. Challenges rely principally on the large size of Cas9 protein and the difficulty to prevent ribonucleoprotein complexes from degrading during the entire formulation and delivery process [183]. Lee et al. used gold NPs (GNP) to develop innovative carriers for the delivery of the entire CRISPR/Cas9 system (named CRISPR-Gold) to restore dystrophin expression by inducing in vivo homologous directed repair (HDR) in *mdx* mice [184]. Authors selected GNP as they can be easily coated with a densely packed layer of DNA and can be internalised by a variety of cell types [221,222]. To obtain complex CRISPR-Gold, the authors coated GNP with a thiol-terminated DNA to efficiently hybridize thiol-terminated donor DNA and trigger its rapid release once into the cytoplasm by disulfide-bond cleavage. Cas9 protein/sgRNA was then adsorbed onto the NPs, then finally covered with PAsp(DET) endosomal disruptive polymer. CRISPR-Gold demonstrated the ability to efficiently deliver in vitro and in vivo both the protein and the nucleic acid of the CRISPR/Cas9 system, through their affinity with the GNP coating of packed layer DNA. Injected simultaneously with cardiotoxin to induce further muscle damage, CRISPR-Gold revealed in *mdx* mice an HDR efficiency up to 18 times higher than CRISPR/Cas9 system itself with 5.4% restoration of the dystrophin gene. Moreover, cryosections of CRISPR-Gold-injected muscles showed a robust dystrophin expression nearly similar to that of wild-type mice muscle and reduced levels of muscle fibrosis, a sign of better tissue health. CRISPR-Gold delivered under clinically relevant conditions (without cardiotoxin) were shown to enhance animal strength and agility in *mdx* mice with HDR efficiency of 1% in the dystrophin gene and minimal off-target genomic damage. Finally, this work evidenced the absence of a broad immune response that could be potentially induced by the Cas9 bacterial protein, suggesting the possible safety of multiple injections of CRISPR-Gold. In conclusion, the authors designed NPs able to bind all CRISPR/Cas9 components but also to intracellularly deliver them through endosomal disruptive and disulphide reduction mechanisms. Complex and innovative CRISPR-Gold has the potential to regenerate wild-type dystrophin to a fully functional level, appearing as a promising treatment for genetic diseases such as DMD.

Recently, Wei et al. efficiently delivered Cas9/sgRNA ribonucleoprotein complexes to muscle, brain, liver and lungs using lipid NPs [183]. By adjusting the molecular components and ratios of lipids, the authors achieved tissue-specific gene editing in liver and lungs of healthy mice C57BL/6J after systemic injection. More interestingly, these nanosystems were evaluated in ΔEx44 DMD mice and were proven to restore dystrophin expression up to ~5% after I.M. administration.

To our knowledge, NPs here presented are the only CRISPR/Cas9 delivery systems using non-viral NPs that have been described for MD applications. Although CRISPR/Cas9 is currently one of the most efficient tools for genome editing, non-viral delivery strategies are needed to improve precise gene correction.

## 5. Limitations In In Vitro and In Vivo Testing of Novel Treatments

**Figure 4:**
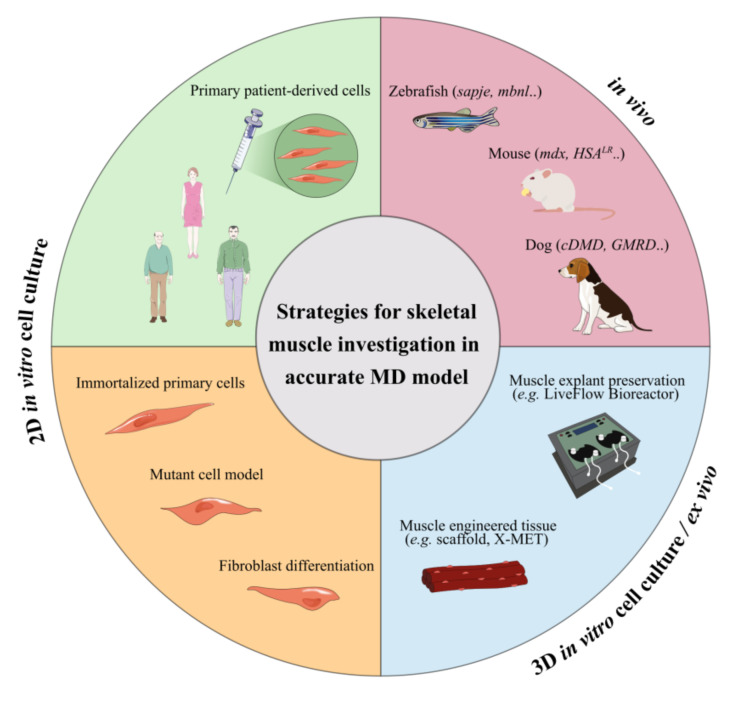
Strategies and accurate models to explore MD treatments. Broad in vitro, ex vivo and in vivo MD models to promote the translation of nanomedicine-based therapies (MD, muscular dystrophies; X-MET, ex vivo-vascularized muscle engineered tissue).

The evaluation of nanomedicine behaviour in appropriate in vitro and in vivo models able to mimic physiology and phenotypes of MDs is an important requirement for their clinical translation. For preliminary studies and screening of different drugs, in vitro and ex vivo models based on immortalised or mutant human and animal cells, muscle preservation systems or tissue engineered constructs have been set-up as alternatives to rare patient cells. In addition, different animal models have been also set up to provide accurate in vivo MD models. An overview of the common used in vitro, ex vivo or in vivo models for testing novel MD treatments is provided in Figure 4.

In vitro studies provide a unique preliminary resource to clarify the potential risk of NPs administration [223]. Primary cells are the most representative cell model for studying the molecular hallmarks of these pathologies as they are directly isolated from the patient’s tissue [224]. However, their use is limited by the poor availability of muscle tissue due to the small samples collected by biopsy and the limited proliferative capacity of the satellite cells isolated from the explanted tissue [225,226,227,228,229]. To overcome these limitations, different strategies have been proposed. Genetic mutation characteristics of the pathology can be exogenously introduced into immortalised cell lines such as HeLa, C2C12 myoblasts or induced pluripotent stem cells (iPS). Even if these experimental models do not reproduce the entire genomic context, these transfected cells express the main features of the pathogenic mechanism, providing a cell-based model for studying the splicing defects of MDs [18,86,230]. Another approach consists in the immortalisation of primary muscle cells by reducing their replicative senescence with the use of telomere shortening, inhibiting the dominant p16 pathway [223,231,232,233]. Skin fibroblasts from patient skin biopsies can also differentiate into multinucleated myotubes by inducing the myogenic regulator factor MyoD [223,224,234,235]. However, these 2D monolayer cultures inaccurately representing in vitro tissue cells and cellular response to therapeutic treatments might be erroneous due to the unnatural microenvironment [236,237]. In this context, 3D culture systems have gained increasing interest as they are able to provide accurate models of organs or tissue physiology and associated disorders. However, they are not exempt from limitations, such as lack of nutrients and oxygen distribution, and accumulation of wastes into the core of 3D culture [236,238,239]. Diverse biomimetic engineered muscle constructs, such as scaffold or organoid cell culture, have demonstrated structural and functional characteristics similar to native muscle, mimicking the tissue complexity [240,241,242]. These skeletal muscle organoids offer an attractive alternative to preliminary in vivo studies for disease modelling and in vitro drug screening.

Ex-vivo models have also reproduced accurate natural environment models for broad applications, overcoming 3D cell culture challenges related to heterogeneous oxygen, nutrient and metabolic waste distribution [237,243,244,245]. One possible approach for muscle investigations is culturing skeletal muscle explants as intact muscle fibres (myofibers) or intact muscles, preserving cell/tissue architecture and its relationship with the surrounding anatomical structures [246]. Moreover, fluid dynamic systems may improve the organ preservation, overcoming the loss of vasculature function by mimicking the physiological flow for nutrient supply and catabolite withdrawal, thus supporting the metabolic activity of the explanted tissues [243,247]. Recently, Carton et al. demonstrated the great benefit in terms of structural preservation obtained by maintaining explanted soleus murine muscle in a bioreactor under dynamic conditions [248]. The progressive structural deterioration of the muscle tissue was markedly slowed, prolonging its preservation up to two days. This innovative system allows experimental testing on the living organism with positive ethical impact, overcoming animal injuries related to therapeutic trial. Bioengineered three-dimensional vascularised skeletal muscle tissue (named X-MET) has also been developed by Carosio et al., using heterogeneous primary cell populations such as myoblasts, fibroblasts and endothelial cells [249]. This vascularised ex-vivo system closely mimicking the cellular complexity of the muscle tissue showed biomechanical properties and activity similar to adult skeletal muscles. Furthermore, X-MET transplanted into damaged muscle has demonstrated interesting properties to restore muscle functionality.

To further investigate therapeutic efficacy, in vivo testing remains the most representative means of study for understanding the complex cellular and tissue mechanisms’ interactions [250,251]. Over the last years, various animal models of MDs have contributed to clarifying the molecular phenotypes involved in these pathologies and investigating drug screening [252,253,254]. Several DMD animal models have been developed by reproducing the deficient dystrophin expression. As previously reported, the *mdx* mouse (BL10-*mdx*) is the most commonly used DMD animal model and is characterised by the presence of a stop codon located in exon 23 that leads to loss of full-length dystrophin whereas smaller isoforms are still expressed [89,255]. Recently, D2-*mdx* mouse models issued from a different genetic background (DBA2/J) have demonstrated a more pronounced phenotype closer to patients [256]. Furthermore, Desguerre et al. described a model of chronic mechanical muscle injury to trigger muscle fibrosis at the same time of dystrophin-deficiency expression in *mdx* hindlimb muscle [257]. DMD canine model (cDMD, GRMD) also carries dystrophin deficit and expresses clinical phenotypes more severely than *mdx* mouse, better aligning with the progressive course of DMD and thereby better translating to humans [252,258,259]. Two zebrafish dystrophin mutants, sapje and sapje-like (sapc/100), carry a mutation in the dystrophin gene, which results in a premature stop codon, thus mimicking muscle dysfunction with a severe phenotype [260,261]. Other less common DMD animal models include *Caenorhabditis elegans*, *Drosophila melanogaster*, feline, rat and pig models [252].

Regarding DM, animal models have been developed by reproducing CUG expansion, MBNL-deficient or CELF-overexpressing phenotypes. Two main mouse models are commonly used for the study of DM1 and DM2: DMSXL mice, carrying ~ 1000–1800 CTG repeats with multisystemic transgene expression, and HSA^LR^ mice that express human skeletal actin (HSA) transcripts containing ~ 250 trinucleotide repeats within the 3′ DMPK UTR with skeletal muscle transgene expression [262,263]. These murine models reproduce the DM1 and DM2 pathogenesis similarly to the human phenotype [264,265,266,267,268]. Two zebrafish models of MBNL loss of function, typical of DM1, have been generated to study the pathological aspects that characterise skeletal and heart muscles in DM [269,270]. Other transgenic animals, such as *Caenorhabditis elegans* and *Drosophila melanogaster* have also been engineered to mimic some characteristics of DM phenotypes [252,262].

Although no animal model is able to completely recapitulate the aspects of the multisystemic phenotypes typical of MDs, their use is required to provide critical in-depth assessment of proof-of-principle concept studies and preclinical experiments. The actual growing interest and knowledge for development of in vitro and ex vivo alternatives to reduce clinical animal experimentation could warrant novel and better methods for assisting in assessment of MDs therapy.

## 6. Future Perspectives

The recent understanding of the pathogenic mechanisms of MDs highlights the urgent need of new and more effective treatments [20,100]. Nanomedicine demonstrated to enhance the therapeutic potential of gene therapy and drug repurposing approaches. As an example, pentamidine-loaded nanomedicines were used to explore the activity of the drug, not only as an anti-leishmaniasis agent but also as an anticancer agent to reduce drug associated toxicity, such as its severe nephrotoxicity [271,272]. Ongoing investigations are aimed at demonstrating the efficacy of this novel formulation to treat DM1 (study in progress).

New therapeutic approaches needed a continuous administration throughout patient’s life, making the biocompatibility and biodegradability of delivery systems a crucial feature to preserve skeletal muscle from additional alterations. As presented in this review, the main advantages of nanosystems rely on their physico-chemical properties, namely composition, size and surface potential that can be modulated to avoid nanocarrier toxicity, and to load specific actives and deliver them in a target site.

To disclose the potential of nanomedicine application to MDs treatment, the gap between in vitro and in vivo testing has to be filled. In addition, to understand the fate of nanosystems once administered to MDs mice, biodistribution studies need to be addressed. To date, only a few investigations reported NPs biodistribution into skeletal muscles through different administration routes as I.V., I.P. and I.M. (tibialis anterior and gastrocnemius muscles) [173,273,274]. After systemic administration, NPs spread into tissues through blood systemic circulation, then, extravasate into the ECM before reaching muscle fibres. It has been suggested that the dense blood capillary network wrapping skeletal myofibers could be favourable to NPs accumulation and distribution following I.V. injection [196]. Hydrodynamic injection is also known to facilitate gene delivery by transient enhancement of the plasma membrane’s permeability [142,275,276]. However, the applied pressure due to the hindrance of the blood flow might cause oedema and inflammation, restricting the translation of this technique to clinic [277].

Overcoming the ECM barrier remains another important goal to improve NPs distribution in skeletal muscle fibres. Surface engineered nanosystems have been designed to actively promote the interaction between nanosystems and cells [278]. The high specificity of antibodies for their corresponding antigen provides a selective and potent approach for therapeutic NPs targeting [279]. As an example, the murine monoclonal antibody (3E10), capable of binding the surface of muscle cells, has been reported to improve active targeting [280,281]. However, no scientific studies on antibody-functionalised NPs have been conducted for skeletal muscle targeting so far. More commonly used, short peptides sequences (*e.g.,* ASSLNIA or SKTFNTHPQSTP) have proved promising as NPs functionalization for specific tissue-targeting [282,283,284]. Several examples of peptides targeting muscle cells have been reported [285,286,287] and association to nanosystems may lead to improved selectivity of NPs for skeletal muscle. Polymeric nanosystems have been functionalised with active targeting agents that preferentially bind active molecules or receptors expressed on the surface of muscle cells. Active targeting-dependent uptake has been demonstrated using PLGA nanocarriers functionalised with a muscle-homing peptide M12 [288]. Biodistribution studies revealed a preferential accumulation of targeted NPs in skeletal muscle cells in *mdx* mice, compared to untargeted nanocarriers, increasing the accumulation of polymeric NPs and enhancing therapeutic efficacy [274].

As presented in this review, nanomedicine holds promise for the development of efficient and safe MD treatments. With the rise in biologic products development, there is an increasing interest for effective biocompatible delivery systems that can be better suited for biologics leading to more effective therapeutic strategies. Further development of nanomedicine in this area is expected to result from complementary expertise in different research fields. Indeed, a multi-disciplinary study in drug discovery, nanomedicine, biotechnology, biology and medicine is key to providing valid and reliable strategies that can offer unprecedented opportunities to MD patients.

## Figures and Tables

**Table 1:** Therapeutic strategies for the treatment of MDs (DMD, Duchenne Muscular Dystrophy; DM, Myotonic Dystrophy; ASO, antisense oligonucleotide; CRISPR, clustered regularly interspaced short palindromic repeats).

Approach	Active Agent	Target	Pathology	Limitations	Development Phase	Reference
**Antisense oligonucleotides**	Etelprisen	mRNA	DMD	Rapid degradation by exonuclease Low cellular uptake Activation of immune systemInflammatory effects	FDA approved (Clin. Trial NCT02255552)	[77]
	Drisapersen				Phase III (Clin. Trial NCT01254019)	[78,79]
	cEt ASO	CUG/CCUG^exp^	DM		Preclinical studies	[80,81]
**CRISPR/Cas9**	Not approved	DNA	DMD	Higher accumulation in proliferating cells than in fully differentiated cells Rapid degradation	Preclinical studies	[82,83,84,85]
	Not approved	CTG^exp^ DNA	DM	Uncompleted repair of protein expression Low transfection efficiency	Preclinical studies	[86]
**Small molecules**	Aminoglycoside antibiotics	Non-sense mutations on mRNA	DMD	Ototoxicity Nephrotoxicity	Preclinical studies	[87,88,89]
	Ataluren	Non-sense mutations on mRNA	DMD	High dosage required	EMA approved (Clin. Trial NCT01826487)	[90,91]
	Pentamidine	CUG^exp^ RNA	DM	Nephrotoxicity Off-label use	Preclinical studies	[18,92]
	Furamidine and erytromicin	CTG^exp^ DNA	DM	Off-label use	Preclinical studies	[93,94,95]
	ISOX and vorinostat	MBNL1-splicing factors	DM1	Off-label use	Preclinical studies	[96]

**Table 2:** Recapitulative table of the diverse described nanosystems tested in vivo for treating MDs (PEI, polyethylenimine; PEG, polyethylene glycol; PLGA, poly(lactic-*co*-glycolic acid); PMMA, poly(methyl methacrylate); NIPAM, N-isopropylacrylamide; PEA, poly(ethylene adipate); PLys, poly(l-lysine); PPE-EA, poly(2-aminoethyl propylene phosphate); PAMAM-OH, hydroxyl-terminated poly(amidoamine); DMPC, L-a-dimyristoylphosphatidylcholine; (C12(EO)23), polyoxyethylene(23) lauryl ether; NPs, nanoparticles; ASO, antisense oligonucleotide; PMO, phosphorodiamidate morpholino oligomer; GA, glatiramer acetate; CRISPR, clustered regularly interspaced short palindromic repeats; I.M., intramuscular injection; I.V., intravenous injection; I.P., intraperitoneal).

Class of Nanocarriers	Nanocarrier Composition	Muscle Pathology	Loaded Molecules	Therapeutic Target	Mouse Model	Advantages and Limitations	Admin. Route	Ref.
**Polymeric**	PEI-PEG	DMD	2′-OMe ASO	Dystrophin pre-mRNA	mdx	(+) high dystrophin-positive fibers increased (+) long term residual efficacy over 6 weeks (-) low general transfection efficiency	I.M.	[163]
	PEI-PEG/PLGA	DMD	2′-OMe ASO	Dystrophin pre-mRNA	mdx	(-) no improvement compared to PEI-PEG-ASO	I.M.	[164]
	PEI-Pluronic^®^	DMD	PMO ASO	Dystrophin pre-mRNA	mdx	(+) dystrophin-positive fibers increased up to 4-fold after I.M. (+) dystrophin-positive fibers increased up to 3-fold in all skeletal muscles after I.V. (+) dystrophin-positive fibers increased up to 5-fold in heart after I.V. (+) low muscle tissue, liver and kidney toxicity (-) mild general transfection efficiency	I.M./I.V.	[165]
		DMD	2′-OMe ASO	Dystrophin pre-mRNA	mdx	(+) dystrophin-positive fibers increased up to 10-fold	I.M.	[166]
	PEG-polycaprolactone PEG-(polylactic acid)	DMD	PMO ASO	Dystrophin pre-mRNA	mdx	(+) dystrophin-positive fibers increased up to 3-fold (+) low muscle tissue toxicity (-) mild general transfection efficiency	I.M.	[167]
	PMMA	DMD	2′-OMe ASO	Dystrophin pre-mRNA	mdx	(+) dystrophin-positive fibers increased up to 7-fold (-) slow biodegradability	I.P.	[168]
	PMMA/NIPAM	DMD	2′-OMe ASO	Dystrophin pre-mRNA	mdx	(+) dystrophin-positive fibers increased up to 4-fold (+) body-wide dystrophin restoration after I.V. (+) exon-skipping level enhanced up to 20-fold (+) long term residual efficacy over 90 days	I.P./I.V.	[169,170]
	PEA	DMD	2′-OMe ASO	Dystrophin pre-mRNA	mdx	(+) dystrophin-positive fibers increased up to 3–10-fold	I.M.	[171]
		DMD	PMO ASO	Dystrophin pre-mRNA	mdx	(+) dystrophin-positive fibers increased up to 3-fold after I.M. (+) body-wide dystrophin-positive fibers increased up to 3-fold after I.V.	I.M./I.V.	[171]
		Muscle atrophy/ DMD	pDNA	Cell nucleus	mdx	(+) transfection efficiency enhanced up to 6-fold	I.M.	[172]
	PLys-PEG	Muscle atrophy	pDNA	Cell nucleus	Balb/c	(+) transfection efficiency enhanced up to 10-fold	I.V.	[173]
	PPE-EA	Muscle atrophy	pDNA	Cell nucleus	Balb/c	(+) transfection efficiency enhanced up to 13-fold (+) long term residual efficacy over 14 days	I.M.	[174]
	Atelocollagen	Muscle atrophy/ DMD	siRNA	Cytoplasm	mdx	(+) higher mass muscle increase	I.M./I.V.	[175]
	PAMAM-OH	Muscle atrophy	Angiotensin (1–7)	Cytoplasm	Balb/c	(+) higher anti-atrophic effects	I.P.	[176]
**Lipidic**	PEG-bubble liposomes	DMD	PMO ASO	Dystrophin pre-mRNA	mdx	(+) dystrophin-positive fibers increased up to 1.5-fold (+) exon-skipping level enhanced up to 5-fold	I.M.	[177]
		DM1	PMO ASO	Clcn1 pre-mRNA	HSA^LR^	(+) increased expression of Clcn1 protein up to 1.4-fold	I.M.	[178]
	Nanolipodendrosomes	DMD	MyoD and GA	Cytoplasm	SW-1	(+) slight mass muscle increase	I.M.	[179]
	Nanoliposomes	DMD	Glucocorticoide	Cell nucleus	mdx	(+) lower inflammatory induced response (+) lower bone catabolic effects	I.V.	[180]
	Hybrid liposomes DMPC and (C_12_(EO)_23_)	DMD	Gentamicin	Ribosomes	mdx	(+) dystrophin-positive fibers increased up to 4-fold (+) lower ototoxicity and nephrotoxicity	I.P.	[181]
	Perfluorocarbon	DMD	Rapamycin	mTORC1 complex	mdx	(+) high muscle strength increase (+) high cardiac contractile performance increase	I.V.	[182]
	Lipid NPs	DMD	CRISPR/Cas9	Dystrophin DNA sequence	ΔEx44	(+) dystrophin expression restored up to 5%	I.M.	[183]
**Inorganic**	Gold	DMD	CRISPR/Cas9	Dystrophin DNA sequence	mdx	(+) HDR in the dystrophin gene enhanced up to 18-fold	I.M.	[184]

## Data Availability

Not applicable.

## References

[1] Shieh P.B. (2013). Muscular dystrophies and other genetic myopathies. Neurol. Clin..

[2] Theadom A., Rodrigues M., Roxburgh R., Balalla S., Higgins C., Bhattacharjee R., Jones K., Krishnamurthi R., Feigin V. (2014). Prevalence of muscular dystrophies: A systematic literature review. Neuroepidemiology.

[3] Mercuri E., Bönnemann C.G., Muntoni F. (2019). Muscular dystrophies. Lancet Lond. Engl..

[4] Carter J.C., Sheehan D.W., Prochoroff A., Birnkrant D.J. (2018). Muscular Dystrophies. Clin. Chest Med..

[5] Johnson N.E. (2019). Myotonic Dystrophies. Continuum.

[6] Meola G. (2013). Clinical aspects, molecular pathomechanisms and management of myotonic dystrophies. Acta Myol..

[7] Messina S., Vita G.L. (2018). Clinical management of Duchenne muscular dystrophy: The state of the art. Neurol. Sci..

[8] Nio Y., Tanaka M., Hirozane Y., Muraki Y., Okawara M., Hazama M., Matsuo T. (2017). Phosphodiesterase 4 inhibitor and phosphodiesterase 5 inhibitor combination therapy has antifibrotic and anti-inflammatory effects in *mdx* mice with Duchenne muscular dystrophy. FASEB J..

[9] Zanotti S., Bragato C., Zucchella A., Maggi L., Mantegazza R., Morandi L., Mora M. (2016). Anti-fibrotic effect of pirfenidone in muscle derived-fibroblasts from Duchenne muscular dystrophy patients. Life Sci..

[10] McDonald C.M., Henricson E.K., Abresch R.T., Duong T., Joyce N.C., Hu F., Clemens P.R., Hoffman E.P., Cnaan A., Gordish-Dressman H. (2018). Long-term effects of glucocorticoids on function, quality of life, and survival in patients with Duchenne Muscular Dystrophy: A prospective cohort study. Lancet.

[11] Kao K.-T., Joseph S., Capaldi N., Brown S., Di Marco M., Dunne J., Horrocks I., Shepherd S., Ahmed S.F., Wong S.C. (2019). Skeletal disproportion in glucocorticoid-treated boys with Duchenne muscular dystrophy. Eur. J. Pediatr..

[12] Mayo A.L., Craven B.C., McAdam L.C., Biggar W.D. (2012). Bone health in boys with Duchenne muscular dystrophy on long-term daily deflazacort therapy. Neuromuscul. Disord..

[13] Ward L.M., Weber D.R. (2019). Growth, pubertal development, and skeletal health in boys with Duchenne muscular dystrophy. Curr. Opin. Endocrinol. Diabetes Obes..

[14] Birnkrant D.J., Bushby K., Bann C.M., Apkon S.D., Blackwell A., Brumbaugh D., Case L.E., Clemens P.R., Hadjiyannakis S., Pandya S. (2018). Diagnosis and management of Duchenne muscular dystrophy, part 1: Diagnosis, and neuromuscular, rehabilitation, endocrine, and gastrointestinal and nutritional management. Lancet Neurol..

[15] Fayssoil A., Lazarus A., Wahbi K., Ogna A., Nardi O., Lofaso F., Clair B., Orlikowski D., Annane D. (2016). Cardiac implantable electronic devices in tracheotomized muscular dystrophy patients: Safety and risks. Int. J. Cardiol..

[16] Bach J.R., Saporito L.R., Shah H.R., Sinquee D. (2014). Decanulation of patients with severe respiratory muscle insufficiency: Efficacy of mechanical insufflation-exsufflation. J. Rehabil. Med..

[17] Warf M.B., Nakamori M., Matthys C.M., Thornton C.A., Berglund J.A. (2009). Pentamidine reverses the splicing defects associated with myotonic dystrophy. Proc. Natl. Acad. Sci. USA.

[18] Konieczny P., Selma-Soriano E., Rapisarda A.S., Fernandez-Costa J.M., Perez-Alonso M., Artero R. (2017). Myotonic dystrophy: Candidate small molecule therapeutics. Drug Discov. Today.

[19] Lee J.E., Bennett C.F., Cooper T.A. (2012). RNase H-mediated degradation of toxic RNA in myotonic dystrophy type 1. Proc. Natl. Acad. Sci. USA.

[20] Verhaart I.E.C., Aartsma-Rus A. (2019). Therapeutic developments for Duchenne muscular dystrophy. Nat. Rev. Neurol..

[21] Zhang Y., Li H., Min Y.L., Sanchez-Ortiz E., Huang J., Mireault A.A., Shelton J.M., Kim J., Mammen P.P.A., Bassel-Duby R. (2020). Enhanced CRISPR-Cas9 correction of Duchenne muscular dystrophy in mice by a self-complementary AAV delivery system. Sci. Adv..

[22] Lo Scrudato M., Poulard K., Sourd C., Tomé S., Klein A.F., Corre G., Huguet A., Furling D., Gourdon G., Buj-Bello A. (2019). Genome editing of expanded CTG repeats within the human DMPK gene reduces nuclear RNA foci in the muscle of DM1 mice. Mol. Ther..

[23] Pedrini I., Gazzano E., Chegaev K., Rolando B., Marengo A., Kopecka J., Fruttero R., Ghigo D., Arpicco S., Riganti C. (2014). Liposomal nitrooxy-doxorubicin: One step over caelyx in drug-resistant human cancer cells. Mol. Pharm..

[24] Autio K.A., Dreicer R., Anderson J., Garcia J.A., Alva A., Hart L.L., Milowsky M.I., Posadas E.M., Ryan C.J., Graf R.P. (2018). Safety and efficacy of BIND-014, a docetaxel nanoparticle targeting prostate-specific membrane antigen for patients with metastatic castration-resistant prostate cancer: A phase 2 clinical trial. JAMA Oncol..

[25] Van der Meel R., Sulheim E., Shi Y., Kiessling F., Mulder W.J.M., Lammers T. (2019). Smart cancer nanomedicine. Nat. Nanotechnol..

[26] Wang-Gillam A., Hubner R.A., Siveke J.T., Von Hoff D.D., Belanger B., de Jong F.A., Mirakhur B., Chen L.-T. (2019). NAPOLI-1 phase 3 study of liposomal irinotecan in metastatic pancreatic cancer: Final overall survival analysis and characteristics of long-term survivors. Eur. J. Cancer.

[27] Hanif S., Muhammad P., Chesworth R., Rehman F.U., Qian R., Zheng M., Shi B. (2020). Nanomedicine-Based Immunotherapy for Central Nervous System Disorders. Acta Pharmacol. Sin..

[28] You L., Wang J., Liu T., Zhang Y., Han X., Wang T., Guo S., Dong T., Xu J., Anderson G.J. (2018). Targeted brain delivery of rabies virus glycoprotein 29-modified deferoxamine-loaded nanoparticles reverses functional deficits in Parkinsonian mice. ACS Nano.

[29] Dos Santos Tramontin N., da Silva S., Arruda R., Ugioni K.S., Canteiro P.B., de Bem Silveira G., Mendes C., Silveira P.C.L., Muller A.P. (2020). Gold nanoparticles treatment reverses brain damage in Alzheimer’s disease model. Mol. Neurobiol..

[30] Pearson R.M., Podojil J.R., Shea L.D., King N.J.C., Miller S.D., Getts D.R. (2019). Overcoming challenges in treating autoimmuntity: Development of tolerogenic immune-modifying nanoparticles. Nanomedicine.

[31] Zhao G., Liu A., Zhang Y., Zuo Z.-Q., Cao Z.-T., Zhang H.-B., Xu C.-F., Wang J. (2019). Nanoparticle-delivered siRNA targeting Bruton’s tyrosine kinase for rheumatoid arthritis therapy. Biomater. Sci..

[32] Horwitz D.A., Bickerton S., Koss M., Fahmy T.M., La Cava A. (2019). Suppression of murine Lupus by CD4+ and CD8+ treg cells induced by T cell-targeted nanoparticles loaded with interleukin-2 and transforming growth factor β. Arthritis Rheumatol..

[33] Fries C.N., Curvino E.J., Chen J.-L., Permar S.R., Fouda G.G., Collier J.H. (2020). Advances in nanomaterial vaccine strategies to address infectious diseases impacting global health. Nat. Nanotechnol..

[34] (2020). Nanomedicine and the COVID-19 vaccines. Nat. Nanotechnol..

[35] Sahin U., Muik A., Derhovanessian E., Vogler I., Kranz L.M., Vormehr M., Baum A., Pascal K., Quandt J., Maurus D. (2020). COVID-19 vaccine BNT162b1 elicits human antibody and TH1 T cell responses. Nature.

[36] Baden L.R., El Sahly H.M., Essink B., Kotloff K., Frey S., Novak R., Diemert D., Spector S.A., Rouphael N., Creech C.B. (2020). Efficacy and safety of the MRNA-1273 SARS-CoV-2 vaccine. N. Engl. J. Med..

[37] Chung Y.H., Beiss V., Fiering S.N., Steinmetz N.F. (2020). COVID-19 vaccine frontrunners and their nanotechnology design. ACS Nano.

[38] Kranz L.M., Diken M., Haas H., Kreiter S., Loquai C., Reuter K.C., Meng M., Fritz D., Vascotto F., Hefesha H. (2016). Systemic RNA delivery to dendritic cells exploits antiviral defence for cancer immunotherapy. Nature.

[39] Xin X., Kumar V., Lin F., Kumar V., Bhattarai R., Bhatt V.R., Tan C., Mahato R.I. (2020). Redox-responsive nanoplatform for codelivery of miR-519c and gemcitabine for pancreatic cancer therapy. Sci. Adv..

[40] Sasso M.S., Lollo G., Pitorre M., Solito S., Pinton L., Valpione S., Bastiat G., Mandruzzato S., Bronte V., Marigo I. (2016). Low dose gemcitabine-loaded lipid nanocapsules target monocytic myeloid-derived suppressor cells and potentiate cancer immunotherapy. Biomaterials.

[41] Lancet J.E., Uy G.L., Cortes J.E., Newell L.F., Lin T.L., Ritchie E.K., Stuart R.K., Strickland S.A., Hogge D., Solomon S.R. (2018). CPX-351 (cytarabine and daunorubicin) liposome for injection versus conventional cytarabine plus daunorubicin in older patients with newly diagnosed secondary acute myeloid leukemia. J. Clin. Oncol..

[42] Salvioni L., Rizzuto M.A., Bertolini J.A., Pandolfi L., Colombo M., Prosperi D. (2019). Thirty years of cancer nanomedicine: Success, frustration, and hope. Cancers.

[43] Gillies A.R., Lieber R.L. (2011). Structure and function of the skeletal muscle extracellular matrix. Muscle Nerve.

[44] Yhee J.Y., Yoon H.Y., Kim H., Jeon S., Hergert P., Im J., Panyam J., Kim K., Nho R.S. (2017). The effects of collagen-rich extracellular matrix on the intracellular delivery of glycol chitosan nanoparticles in human lung fibroblasts. Int. J. Nanomed..

[45] Sleboda D.A., Stover K.K., Roberts T.J. (2020). Diversity of extracellular matrix morphology in vertebrate skeletal muscle. J. Morphol..

[46] Engin A.B., Nikitovic D., Neagu M., Henrich-Noack P., Docea A.O., Shtilman M.I., Golokhvast K., Tsatsakis A.M. (2017). Mechanistic understanding of nanoparticles’ interactions with extracellular matrix: The cell and immune system. Part. Fibre Toxicol..

[47] Stylianopoulos T., Poh M.Z., Insin N., Bawendi M.G., Fukumura D., Munn L.L., Jain R.K. (2010). Diffusion of particles in the extracellular matrix: The effect of repulsive electrostatic interactions. Biophys. J..

[48] Zhigaltsev I.V., Belliveau N., Hafez I., Leung A.K.K., Huft J., Hansen C., Cullis P.R. (2012). Bottom-up design and synthesis of limit size lipid nanoparticle systems with aqueous and triglyceride cores using millisecond microfluidic mixing. Langmuir.

[49] Evers M.J.W., Kulkarni J.A., van der Meel R., Cullis P.R., Vader P., Schiffelers R.M. (2018). State-of-the-art design and rapid-mixing production techniques of lipid nanoparticles for nucleic acid delivery. Small Methods.

[50] Feng J., Markwalter C.E., Tian C., Armstrong M., Prud’homme R.K. (2019). Translational formulation of nanoparticle therapeutics from laboratory discovery to clinical scale. J. Transl. Med..

[51] Ebner D.C., Bialek P., El-Kattan A.F., Ambler C.M., Tu M. (2015). Strategies for skeletal muscle targeting in drug discovery. Curr. Pharm. Des..

[52] Mah J.K., Korngut L., Fiest K.M., Dykeman J., Day L.J., Pringsheim T., Jette N. (2015). A Systematic review and meta-analysis on the epidemiology of the muscular dystrophies. Can. J. Neurol. Sci..

[53] Nascimento Osorio A., Medina Cantillo J., Camacho Salas A., Madruga Garrido M., Vilchez Padilla J.J. (2019). Consensus on the diagnosis, treatment and follow-up of patients with Duchenne muscular dystrophy. Neurologia.

[54] Koenig M., Hoffman E.P., Bertelson C.J., Monaco A.P., Feener C., Kunkel L.M. (1987). Complete cloning of the Duchenne muscular dystrophy (DMD) cDNA and preliminary genomic organization of the DMD gene in normal and affected individuals. Cell.

[55] Zhu J.F., Liu H.H., Zhou T., Tian L. (2013). Novel mutation in exon 56 of the dystrophin gene in a child with Duchenne muscular dystrophy. Int. J. Mol. Med..

[56] Muntoni F., Torelli S., Ferlini A. (2003). Dystrophin and mutations: One gene, several proteins, multiple phenotypes. Lancet Neurol..

[57] Koenig M., Beggs A.H., Moyer M., Scherpf S., Heindrich K., Bettecken T., Meng G., Müller C.R., Lindlöf M., Kaariainen H. (1989). The molecular basis for Duchenne versus Becker muscular dystrophy: Correlation of severity with type of deletion. Am. J. Hum. Genet..

[58] Miyatake S., Mizobe Y., Takizawa H., Hara Y., Yokota T., Takeda S., Aoki Y. (2018). Exon skipping therapy using phosphorodiamidate morpholino oligomers in the *mdx*52 mouse model of Duchenne muscular dystrophy. Methods Mol. Biol..

[59] Zhu P., Wu F., Mosenson J., Zhang H., He T.-C., Wu W.-S. (2017). CRISPR/Cas9-mediated genome editing corrects dystrophin mutation in skeletal muscle stem cells in a mouse model of muscle dystrophy. Mol. Ther. Nucleic Acids.

[60] Bird T.D., Adam M.P., Ardinger H.H., Pagon R.A., Wallace S.E., Bean L.J., Stephens K., Amemiya A. (1993–2020). Myotonic Dystrophy Type 1. GeneReviews^®^.

[61] Thornton C.A. (2014). Myotonic dystrophy. Neurol. Clin..

[62] Turner C., Hilton-Jones D. (2010). The myotonic dystrophies: Diagnosis and management. J. Neurol. Neurosurg. Psychiatry.

[63] Lee J.E., Cooper T.A. (2009). Pathogenic mechanisms of myotonic dystrophy. Biochem. Soc. Trans..

[64] De Temmerman N., Sermon K., Seneca S., De Rycke M., Hilven P., Lissens W., Van Steirteghem A., Liebaers I. (2004). Intergenerational instability of the expanded CTG repeat in the DMPK gene: Studies in human gametes and preimplantation embryos. Am. J. Hum. Genet..

[65] Malatesta M., Cardani R., Pellicciari C., Meola G. (2014). RNA transcription and maturation in skeletal muscle cells are similarly impaired in myotonic dystrophy and sarcopenia: The ultrastructural evidence. Front. Aging Neurosci..

[66] Nakamori M., Sobczak K., Puwanant A., Welle S., Eichinger K., Pandya S., Dekdebrun J., Heatwole C.R., McDermott M.P., Chen T. (2013). Splicing biomarkers of disease severity in myotonic dystrophy. Ann. Neurol..

[67] Miller J.W., Urbinati C.R., Teng-Umnuay P., Stenberg M.G., Byrne B.J., Thornton C.A., Swanson M.S. (2000). Recruitment of human muscleblind proteins to (CUG)(n) expansions associated with myotonic dystrophy. EMBO J..

[68] Meola G., Cardani R. (2015). Myotonic dystrophies: An update on clinical aspects, genetic, pathology, and molecular pathomechanisms. Biochim. Biophys. Acta.

[69] Paul S., Dansithong W., Kim D., Rossi J., Webster N.J., Comai L., Reddy S. (2006). Interaction of musleblind, CUG-BP1 and hnRNP H proteins in DM1-associated aberrant IR splicing. EMBO J..

[70] Perdoni F., Malatesta M., Cardani R., Giagnacovo M., Mancinelli E., Meola G., Pellicciari C. (2009). RNA/MBNL1-containing foci in myoblast nuclei from patients affected by myotonic dystrophy type 2: An immunocytochemical study. Eur. J. Histochem..

[71] Mulders S.A.M., van den Broek W.J.A.A., Wheeler T.M., Croes H.J.E., van Kuik-Romeijn P., de Kimpe S.J., Furling D., Platenburg G.J., Gourdon G., Thornton C.A. (2009). Triplet-repeat oligonucleotide-mediated reversal of RNA toxicity in myotonic dystrophy. Proc. Natl. Acad. Sci. USA.

[72] Sardone V., Zhou H., Muntoni F., Ferlini A., Falzarano M.S. (2017). Antisense oligonucleotide-based therapy for neuromuscular disease. Molecules.

[73] Kanadia R.N., Shin J., Yuan Y., Beattie S.G., Wheeler T.M., Thornton C.A., Swanson M.S. (2006). Reversal of RNA missplicing and myotonia after muscleblind overexpression in a mouse poly(CUG) model for myotonic dystrophy. Proc. Natl. Acad. Sci. USA.

[74] Arambula J.F., Ramisetty S.R., Baranger A.M., Zimmerman S.C. (2009). A simple ligand that selectively targets CUG trinucleotide repeats and inhibits MBNL protein binding. Proc. Natl. Acad. Sci. USA.

[75] Childs-Disney J.L., Hoskins J., Rzuczek S.G., Thornton C.A., Disney M.D. (2012). Rationally designed small molecules targeting the RNA that causes myotonic dystrophy type 1 are potently bioactive. ACS Chem. Biol..

[76] Udd B., Krahe R. (2012). The myotonic dystrophies: Molecular, clinical, and therapeutic challenges. Lancet Neurol..

[77] Mendell J.R., Rodino-Klapac L.R., Sahenk Z., Roush K., Bird L., Lowes L.P., Alfano L., Gomez A.M., Lewis S., Kota J. (2013). Eteplirsen for the treatment of Duchenne muscular dystrophy. Ann. Neurol..

[78] Goemans N., Mercuri E., Belousova E., Komaki H., Dubrovsky A., Mcdonald C.M., Kraus J.E., Lourbakos A., Lin Z., Campion G. (2018). A randomized placebo-controlled phase 3 trial of an antisense oligonucleotide, drisapersen, in Duchenne muscular dystrophy. Neuromuscul. Disord..

[79] Goemans N.M., Tulinius M., van den Akker J.T., Burm B.E., Ekhart P.F., Heuvelmans N., Holling T., Janson A.A., Platenburg G.J., Sipkens J.A. (2011). Systemic administration of PRO051 in Duchenne’s muscular dystrophy. N. Engl. J. Med..

[80] Pandey S.K., Wheeler T.M., Justice S.L., Kim A., Younis H.S., Gattis D., Jauvin D., Puymirat J., Swayze E.E., Freier S.M. (2015). Identification and characterization of modified antisense oligonucleotides targeting DMPK in mice and nonhuman primates for the treatment of myotonic dystrophy type 1s. J. Pharmacol. Exp. Ther..

[81] Klein A.F., Varela M.A., Arandel L., Holland A., Naouar N., Arzumanov A., Seoane D., Revillod L., Bassez G., Ferry A. (2019). Peptide-conjugated oligonucleotides evoke long-lasting myotonic dystrophy correction in patient-derived cells and mice. J. Clin. Investig..

[82] Min Y.L., Li H., Rodriguez-Caycedo C., Mireault A.A., Huang J., Shelton J.M., McAnally J.R., Amoasii L., Mammen P.P.A., Bassel-Duby R. (2019). CRISPR-Cas9 corrects Duchenne muscular dystrophy exon 44 deletion mutations in mice and human cells. Sci. Adv..

[83] Amoasii L., Hildyard J.C.W., Li H., Sanchez-Ortiz E., Mireault A., Caballero D., Harron R., Stathopoulou T.-R., Massey C., Shelton J.M. (2018). Gene editing restores dystrophin expression in a canine model of Duchenne muscular dystrophy. Science.

[84] Hotta A. (2015). Genome editing gene therapy for Duchenne muscular dystrophy. J. Neuromuscul. Dis..

[85] Amoasii L., Long C., Li H., Mireault A.A., Shelton J.M., Sanchez-Ortiz E., Mcanally J.R., Bhattacharyya S., Schmidt F., Grimm D. (2017). Single-cut genome editing restores dystrophin expression in a new mouse model of muscular dystrophy. Sci. Transl. Med..

[86] Dastidar S., Ardui S., Singh K., Majumdar D., Nair N., Fu Y., Reyon D., Samara E., Gerli M.F.M., Klein A.F. (2018). Efficient CRISPR/Cas9-mediated editing of trinucleotide repeat expansion in myotonic dystrophy patient-derived iPS and myogenic cells. Nucleic Acids Res..

[87] Wagner K.R., Hamed S., Hadley D.W., Gropman A.L., Burstein A.H., Escolar D.M., Hoffman E.P., Fischbeck K.H. (2001). Gentamicin treatment of Duchenne and Becker muscular dystrophy due to nonsense mutations. Ann. Neurol..

[88] Barton-Davis E.R., Cordier L., Shoturma D.I., Leland S.E., Sweeney H.L. (1999). Aminoglycoside antibiotics restore dystrophin function to skeletal muscles of *mdx* mice. J. Clin. Investig..

[89] Vitiello L., Tibaudo L., Pegoraro E., Bello L., Canton M. (2019). Teaching an old molecule new tricks: Drug repositioning for Duchenne muscular dystrophy. Int. J. Mol. Sci..

[90] Welch E.M., Barton E.R., Zhuo J., Tomizawa Y., Friesen W.J., Trifillis P., Paushkin S., Patel M., Trotta C.R., Hwang S. (2007). PTC124 targets genetic disorders caused by nonsense mutations. Nature.

[91] McDonald C.M., Campbell C., Torricelli R.E., Finkel R.S., Flanigan K.M., Goemans N., Heydemann P., Kaminska A., Kirschner J., Muntoni F. (2017). Ataluren in patients with nonsense mutation Duchenne muscular dystrophy (ACT DMD): A multicentre, randomised, double-blind, placebo-controlled, phase 3 trial. Lancet.

[92] Siboni R.B., Bodner M.J., Khalifa M.M., Docter A.G., Choi J.Y., Nakamori M., Haley M.M., Berglund J.A. (2015). Biological efficacy and toxicity of diamidines in myotonic dystrophy type 1 models. J. Med. Chem..

[93] Jenquin J.R., Coonrod L.A., Silverglate Q.A., Pellitier N.A., Hale M.A., Xia G., Nakamori M., Berglund J.A. (2018). Furamidine rescues myotonic dystrophy type I associated mis-splicing through multiple mechanisms. ACS Chem. Biol..

[94] Reddy K., Jenquin J.R., Cleary J.D., Berglund J.A. (2019). Mitigating RNA toxicity in myotonic dystrophy using small molecules. Int. J. Mol. Sci..

[95] Jenquin J.R., Yang H., Huigens III R.W., Nakamori M., Berglund J.A. (2019). Combination treatment of erythromycin and furamidine provides additive and synergistic rescue of mis-splicing in myotonic dystrophy type 1 models. ACS Pharmacol. Transl. Sci..

[96] Zhang F., Bodycombe N.E., Haskell K.M., Sun Y.L., Wang E.T., Morris C.A., Jones L.H., Wood L.D., Pletcher M.T. (2017). A flow cytometry-based screen identifies MBNL1 modulators that rescue splicing defects in myotonic dystrophy type I. Hum. Mol. Genet..

[97] Naldini L. (2015). Gene therapy returns to centre stage. Nature.

[98] Fischer A., Hacein-Bey-Abina S., Cavazzana-Calvo M. (2010). 20 years of gene therapy for SCID. Nat. Immunol..

[99] Dunbar C.E., High K.A., Joung J.K., Kohn D.B., Ozawa K., Sadelain M. (2018). Gene therapy comes of age. Science.

[100] Smith R.A., Miller T.M., Yamanaka K., Monia B.P., Condon T.P., Hung G., Lobsiger C.S., Ward C.M., McAlonis-Downes M., Wei H. (2006). Antisense oligonucleotide therapy for neurodegenerative disease. J. Clin. Investig..

[101] Liang X.H., Sun H., Nichols J.G., Crooke S.T. (2017). RNase H1-dependent antisense oligonucleotides are robustly active in directing RNA cleavage in both the cytoplasm and the nucleus. Mol. Ther..

[102] Geary R.S., Norris D., Yu R., Bennett C.F. (2015). Pharmacokinetics, biodistribution and cell uptake of antisense oligonucleotides. Adv. Drug Deliv. Rev..

[103] Liang X.-H., Shen W., Sun H., Kinberger G.A., Prakash T.P., Nichols J.G., Crooke S.T. (2016). Hsp90 protein interacts with phosphorothioate oligonucleotides containing hydrophobic 2′-modifications and enhances antisense activity. Nucleic Acids Res..

[104] He X.-Y., Wang J., Lu D.-D., Wang S.-Q. (2018). Synthesis and antisense properties of 2′β-F-arabinouridine modified oligonucleotides with 4′-C-OMe substituent. Molecules.

[105] Hagedorn P.H., Pontoppidan M., Bisgaard T.S., Berrera M., Dieckmann A., Ebeling M., Møller M.R., Hudlebusch H., Jensen M.L., Hansen H.F. (2018). Identifying and avoiding off-target effects of RNase H-dependent antisense oligonucleotides in mice. Nucleic Acids Res..

[106] Bosgra S., Sipkens J., De Kimpe S., Den Besten C., Datson N., Van Deutekom J. (2019). The pharmacokinetics of 2′-O-methyl phosphorothioate antisense oligonucleotides: Experiences from developing exon skipping therapies for Duchenne muscular dystrophy. Nucleic Acid Ther..

[107] Hara Y., Mizobe Y., Miyatake S., Takizawa H., Nagata T., Yokota T., Takeda S.I., Aoki Y. (2018). Exon skipping using antisense oligonucleotides for laminin-alpha2-deficient muscular dystrophy. Methods Mol. Biol..

[108] Sicinski P., Geng Y., Ryder-Cook A.S., Barnard E.A., Darlison M.G., Barnard P.J. (1989). The molecular basis of muscular dystrophy in the *mdx* mouse: A point mutation. Science.

[109] Kinali M., Arechavala-Gomeza V., Feng L., Cirak S., Hunt D., Adkin C., Guglieri M., Ashton E., Abbs S., Nihoyannopoulos P. (2009). Local restoration of dystrophin expression with the morpholino oligomer AVI-4658 in Duchenne muscular dystrophy: A single-blind, placebo-controlled, dose-escalation, proof-of-concept study. Lancet Neurol..

[110] Watanabe N., Nagata T., Satou Y., Masuda S., Saito T., Kitagawa H., Komaki H., Takagaki K., Takeda S. (2018). NS-065/NCNP-01: An antisense oligonucleotide for potential treatment of exon 53 skipping in Duchenne muscular dystrophy. Mol. Ther. Nucleic Acids.

[111] Gao Z., Cooper T.A. (2013). Antisense oligonucleotides: Rising stars in eliminating RNA toxicity in myotonic dystrophy. Hum. Gene Ther..

[112] Huguet A., Medja F., Nicole A., Vignaud A., Guiraud-Dogan C., Ferry A., Decostre V., Hogrel J.Y., Metzger F., Hoeflich A. (2012). Molecular, physiological, and motor performance defects in DMSXL mice carrying >1,000 CTG repeats from the human DM1 locus. PLoS Genet..

[113] Wheeler T.M., Leger A.J., Pandey S.K., MacLeod A.R., Nakamori M., Cheng S.H., Wentworth B.M., Bennett C.F., Thornton C.A. (2012). Targeting nuclear RNA for in vivo correction of myotonic dystrophy. Nature.

[114] Jauvin D., Chrétien J., Pandey S.K., Martineau L., Revillod L., Bassez G., Lachon A., McLeod A.R., Gourdon G., Wheeler T.M. (2017). Targeting DMPK with antisense oligonucleotide improves muscle strength in myotonic dystrophy type 1 mice. Mol. Ther. Nucleic Acids.

[115] Ousterout D.G., Kabadi A.M., Thakore P.I., Majoros W.H., Reddy T.E., Gersbach C.A. (2015). Multiplex CRISPR/Cas9-based genome editing for correction of dystrophin mutations that cause Duchenne muscular dystrophy. Nat. Commun..

[116] Ousterout D.G., Kabadi A.M., Thakore P.I., Perez-Pinera P., Brown M.T., Majoros W.H., Reddy T.E., Gersbach C.A. (2015). Correction of dystrophin expression in cells from Duchenne Muscular Dystrophy patients through genomic excision of exon 51 by Zinc Finger Nucleases. Mol. Ther..

[117] Ousterout D.G., Perez-Pinera P., Thakore P.I., Kabadi A.M., Brown M.T., Qin X., Fedrigo O., Mouly V., Tremblay J.P., Gersbach C.A. (2013). Reading frame correction by targeted genome editing restores dystrophin expression in cells from Duchenne Muscular Dystrophy patients. Mol. Ther..

[118] Doudna J.A., Charpentier E. (2014). Genome editing. The new frontier of genome engineering with CRISPR-Cas9. Science.

[119] Nance M.E., Hakim C.H., Yang N.N., Duan D. (2018). Nanotherapy for Duchenne muscular dystrophy. Wiley Interdiscip. Rev. Nanomed. Nanobiotechnol..

[120] Amitai G., Sorek R. (2016). CRISPR-Cas adaptation: Insights into the mechanism of action. Nat. Rev. Microbiol..

[121] Kleinstiver B.P., Prew M.S., Tsai S.Q., Topkar V.V., Nguyen N.T., Zheng Z., Gonzales A.P.W., Li Z., Peterson R.T., Yeh J.-R.J. (2015). Engineered CRISPR-Cas9 nucleases with altered PAM specificities. Nature.

[122] Wang Y., Hao L., Wang H., Santostefano K., Thapa A., Cleary J., Li H., Guo X., Terada N., Ashizawa T. (2018). Therapeutic genome editing for myotonic dystrophy type 1 using CRISPR/Cas9. Mol. Ther..

[123] Naso M.F., Tomkowicz B., Perry W.L., Strohl W.R. (2017). Adeno-associated virus (AAV) as a vector for gene therapy. BioDrugs.

[124] Komor A.C., Kim Y.B., Packer M.S., Zuris J.A., Liu D.R. (2016). Programmable editing of a target base in genomic DNA without double-stranded DNA cleavage. Nature.

[125] Gaudelli N.M., Komor A.C., Rees H.A., Packer M.S., Badran A.H., Bryson D.I., Liu D.R. (2017). Programmable base editing of A•T to G•C in genomic DNA without DNA cleavage. Nature.

[126] Abudayyeh O.O., Gootenberg J.S., Franklin B., Koob J., Kellner M.J., Ladha A., Joung J., Kirchgatterer P., Cox D.B.T., Zhang F. (2019). A Cytosine deaminase for programmable single-base RNA editing. Science.

[127] Ryu S.-M., Koo T., Kim K., Lim K., Baek G., Kim S.-T., Kim H.S., Kim D.-E., Lee H., Chung E. (2018). Adenine base editing in mouse embryos and an adult mouse model of Duchenne Muscular Dystrophy. Nat Biotechnol.

[128] Jiang T., Henderson J.M., Coote K., Cheng Y., Valley H.C., Zhang X.-O., Wang Q., Rhym L.H., Cao Y., Newby G.A. (2020). Chemical modifications of adenine base editor mRNA and guide RNA expand its application scope. Nat Commun.

[129] Anzalone A.V., Randolph P.B., Davis J.R., Sousa A.A., Koblan L.W., Levy J.M., Chen P.J., Wilson C., Newby G.A., Raguram A. (2019). Search-and-replace genome editing without double-strand breaks or donor DNA. Nature.

[130] Pushpakom S., Iorio F., Eyers P.A., Escott K.J., Hopper S., Wells A., Doig A., Guilliams T., Latimer J., McNamee C. (2019). Drug repurposing: Progress, challenges and recommendations. Nat. Rev. Drug Discov..

[131] Knapp S. (2018). New opportunities for kinase drug repurposing and target discovery. Br. J. Cancer.

[132] Wittenstein A., Caspi M., David Y., Shorer Y., Nadar-Ponniah P.T., Rosin-Arbesfeld R. (2019). Serum starvation enhances nonsense mutation readthrough. J. Mol. Med..

[133] Chowdhury H.M., Siddiqui M.A., Kanneganti S., Sharmin N., Chowdhury M.W., Nasim M.T. (2018). Aminoglycoside-mediated promotion of translation readthrough occurs through a non-stochastic mechanism that competes with translation termination. Hum. Mol. Genet..

[134] Shimizu-Motohashi Y., Komaki H., Motohashi N., Takeda S., Yokota T., Aoki Y. (2019). Restoring dystrophin expression in Duchenne muscular dystrophy: Current status of therapeutic approaches. J. Pers. Med..

[135] Hayward R.S., Harding J., Molloy R., Land L., Longcroft-Neal K., Moore D., Ross J.D.C. (2018). Adverse effects of a single dose of gentamicin in adults: A systematic review. Br. J. Clin. Pharmacol..

[136] Friesen W.J., Johnson B., Sierra J., Zhuo J., Vazirani P., Xue X., Tomizawa Y., Baiazitov R., Morrill C., Ren H. (2018). The minor gentamicin complex component, X2, is a potent premature stop codon readthrough molecule with therapeutic potential. PLoS ONE.

[137] Gonzalez À.L., Konieczny P., Llamusi B., Delgado-Pinar E., Borrell J.I., Teixidó J., García-España E., Pérez-Alonso M., Estrada-Tejedor R., Artero R. (2017). In silico discovery of substituted pyrido[2,3-d] pyrimidines and pentamidine-like compounds with biological activity in myotonic dystrophy models. PLoS ONE.

[138] Díaz M.V., Miranda M.R., Campos-Estrada C., Reigada C., Maya J.D., Pereira C.A., López-Muñoz R. (2014). Pentamidine exerts in vitro and in vivo anti Trypanosoma cruzi activity and inhibits the polyamine transport in Trypanosoma cruzi. Acta Trop..

[139] Coonrod L.A., Nakamori M., Wang W., Carrell S., Cameron L., Bodner M.J., Siboni R.B., Docter A.G., Haley M.M., Charles A. (2013). Reducing levels of toxic RNA with small molecules. ACS Chem. Biol..

[140] Theocharis A.D., Skandalis S.S., Gialeli C., Karamanos N.K. (2016). Extracellular matrix structure. Adv. Drug Deliv. Rev..

[141] Zhang G., Budker V., Williams P., Subbotin V., Wolff J.A. (2001). Efficient expression of naked DNA delivered intraarterially to limb muscles of nonhuman primates. Hum. Gene Ther..

[142] Hagstrom J.E., Hegge J., Zhang G., Noble M., Budker V., Lewis D.L., Herweijer H., Wolff J.A. (2004). A facile nonviral method for delivering genes and siRNAs to skeletal muscle of mammalian limbs. Mol. Ther..

[143] Gref R., Minamitake Y., Peracchia M.T., Trubetskoy V., Torchilin V., Langer R. (1994). Biodegradable long-circulating polymeric nanospheres. Science.

[144] Longmire M., Choyke P.L., Kobayashi H. (2008). Clearance properties of nano-sized particles and molecules as imaging agents: Considerations and caveats. Nanomedicine.

[145] Gustafson H.H., Holt-Casper D., Grainger D.W., Ghandehari H. (2015). Nanoparticle uptake: The phagocyte problem. Nano Today.

[146] Blanco E., Shen H., Ferrari M. (2015). Principles of nanoparticle design for overcoming biological barriers to drug delivery. Nat. Biotechnol..

[147] Nguyen V.H., Lee B.-J. (2017). Protein corona: A new approach for nanomedicine design. Int. J. Nanomed..

[148] Tenzer S., Docter D., Kuharev J., Musyanovych A., Fetz V., Hecht R., Schlenk F., Fischer D., Kiouptsi K., Reinhardt C. (2013). Rapid formation of plasma protein corona critically affects nanoparticle pathophysiology. Nat. Nanotechnol..

[149] Stano A., Nembrini C., Swartz M.A., Hubbell J.A., Simeoni E. (2012). Nanoparticle size influences the magnitude and quality of mucosal immune responses after intranasal immunization. Vaccine.

[150] Hoshyar N., Gray S., Han H., Bao G. (2016). The effect of nanoparticle size on in vivo pharmacokinetics and cellular interaction. Nanomedicine.

[151] Vlasova I.I., Kapralov A.A., Michael Z.P., Burkert S.C., Shurin M.R., Star A., Shvedova A.A., Kagan V.E. (2016). Enzymatic oxidative biodegradation of nanoparticles: Mechanisms, significance and applications. Toxicol. Appl. Pharmacol..

[152] Tidball J.G., Welc S.S., Wehling-Henricks M. (2018). Immunobiology of inherited muscular dystrophies. Compr. Physiol..

[153] Smoak M.M., Mikos A.G. (2020). Advances in biomaterials for skeletal muscle engineering and obstacles still to overcome. Mater. Today Bio.

[154] Evers M.M., Toonen L.J.A., van Roon-Mom W.M.C. (2015). Antisense oligonucleotides in therapy for neurodegenerative disorders. Adv. Drug Deliv. Rev..

[155] Kauffman K.J., Webber M.J., Anderson D.G. (2016). Materials for non-viral intracellular delivery of messenger RNA therapeutics. J. Control. Release.

[156] Parlea L., Puri A., Kasprzak W., Bindewald E., Zakrevsky P., Satterwhite E., Joseph K., Afonin K.A., Shapiro B.A. (2016). Cellular delivery of RNA nanoparticles. ACS Comb. Sci..

[157] Wahane A., Waghmode A., Kapphahn A., Dhuri K., Gupta A., Bahal R. (2020). Role of lipid-based and polymer-based non-viral vectors in nucleic acid delivery for next-generation gene therapy. Molecules.

[158] Mastria E.M., Cai L.Y., Kan M.J., Li X., Schaal J.L., Fiering S., Gunn M.D., Dewhirst M.W., Nair S.K., Chilkoti A. (2018). Nanoparticle formulation improves doxorubicin efficacy by enhancing host antitumor immunity. J. Control. Release.

[159] Yu A.-M., Choi Y.H., Tu M.-J. (2020). RNA drugs and RNA targets for small molecules: Principles, progress, and challenges. Pharmacol. Rev..

[160] Givens B.E., Naguib Y.W., Geary S.M., Devor E.J., Salem A.K. (2018). Nanoparticle based delivery of CRISPR/Cas9 genome editing therapeutics. AAPS J..

[161] Liu C., Zhang L., Liu H., Cheng K. (2017). Delivery strategies of the CRISPR-Cas9 gene-editing system for therapeutic applications. J. Control. Release.

[162] Min Y.-L., Bassel-Duby R., Olson E.N. (2019). CRISPR correction of Duchenne muscular dystrophy. Annu. Rev. Med..

[163] Williams J.H., Schray R.C., Sirsi S.R., Lutz G.J. (2008). Nanopolymers improve delivery of exon skipping oligonucleotides and concomitant dystrophin expression in skeletal muscle of *mdx* mice. BMC Biotechnol..

[164] Sirsi S.R., Schray R.C., Wheatley M.A., Lutz G.J. (2009). Formulation of polylactide-co-glycolic acid nanospheres for encapsulation and sustained release of poly(ethylene imine)-poly(ethylene glycol) copolymers complexed to oligonucleotides. J. Nanobiotechnol..

[165] Wang M., Wu B., Lu P., Cloer C., Tucker J.D., Lu Q. (2013). Polyethylenimine-modified Pluronics (PCMs) improve morpholino oligomer delivery in cell culture and dystrophic *mdx* mice. Mol. Ther..

[166] Wang M., Wu B., Lu P., Tucker J.D., Milazi S., Shah S.N., Lu Q.L. (2014). Pluronic–PEI copolymers enhance exon-skipping of 2′-O-methyl phosphorothioate oligonucleotide in cell culture and dystrophic *mdx* mice. Gene Ther..

[167] Kim Y., Tewari M., Pajerowski J.D., Cai S., Sen S., Williams J., Sirsi S., Lutz G., Discher D.E. (2009). Polymersome delivery of siRNA and antisense oligonucleotides. J. Control. Release.

[168] Rimessi P., Sabatelli P., Fabris M., Braghetta P., Bassi E., Spitali P., Vattemi G., Tomelleri G., Mari L., Perrone D. (2009). Cationic PMMA nanoparticles bind and deliver antisense oligoribonucleotides allowing restoration of dystrophin expression in the *mdx* mouse. Mol. Ther..

[169] Ferlini A., Sabatelli P., Fabris M., Bassi E., Falzarano S., Vattemi G., Perrone D., Gualandi F., Maraldi N.M., Merlini L. (2010). Dystrophin restoration in skeletal, heart and skin arrector pili smooth muscle of *mdx* mice by ZM2 NP–AON complexes. Gene Ther..

[170] Bassi E., Falzarano S., Fabris M., Gualandi F., Merlini L., Vattemi G., Perrone D., Marchesi E., Sabatelli P., Sparnacci K. (2012). Persistent dystrophin protein restoration 90 days after a course of intraperitoneally administered naked 2′OMePS AON and ZM2 NP-AON complexes in *mdx* Mice. J. Biomed. Biotechnol..

[171] Wang M., Wu B., Tucker J.D., Bollinger L.E., Lu P., Lu Q. (2016). Poly(ester amine) composed of polyethylenimine and Pluronic enhance delivery of antisense oligonucleotides in vitro and in dystrophic *mdx* mice. Mol. Ther. Nucleic Acids.

[172] Wang M., Tucker J.D., Lu P., Wu B., Cloer C., Lu Q. (2012). Tris[2-(acryloyloxy)ethyl]isocyanurate cross-linked low-molecular-weight polyethylenimine as gene delivery carriers in cell culture and dystrophic *mdx* mice. Bioconjug. Chem..

[173] Itaka K., Osada K., Morii K., Kim P., Yun S.-H., Kataoka K. (2010). Polyplex nanomicelle promotes hydrodynamic gene introduction to skeletal muscle. J. Control. Release.

[174] Wang J., Zhang P.-C., Mao H.-Q., Leong K.W. (2002). Enhanced gene expression in mouse muscle by sustained release of plasmid DNA using PPE-EA as a carrier. Gene Ther..

[175] Kinouchi N., Ohsawa Y., Ishimaru N., Ohuchi H., Sunada Y., Hayashi Y., Tanimoto Y., Moriyama K., Noji S. (2008). Atelocollagen-mediated local and systemic applications of myostatin-targeting siRNA increase skeletal muscle mass. Gene Ther..

[176] Márquez-Miranda V., Abrigo J., Rivera J.C., Araya-Duran I., Aravena J., Simon F., Pacheco N., Gonzalez-Nilo F.D., Cabello-Verrugio C. (2017). The complex of PAMAM-OH dendrimer with angiotensin (1-7) prevented the disuse-induced skeletal muscle atrophy in mice. Int. J. Nanomed..

[177] Negishi Y., Ishii Y., Shiono H., Akiyama S., Sekine S., Kojima T., Mayama S., Kikuchi T., Hamano N., Endo-Takahashi Y. (2014). Bubble liposomes and ultrasound exposure improve localized morpholino oligomer delivery into the skeletal muscles of dystrophic *mdx* mice. Mol. Pharm..

[178] Koebis M., Kiyatake T., Yamaura H., Nagano K., Higashihara M., Sonoo M., Hayashi Y., Negishi Y., Endo-Takahashi Y., Yanagihara D. (2013). Ultrasound-enhanced delivery of morpholino with bubble liposomes ameliorates the myotonia of myotonic dystrophy model mice. Sci. Rep..

[179] Afzal E., Zakeri S., Keyhanvar P., Bagheri M., Mahjoubi P., Asadian M., Omoomi N., Dehqanian M., Ghalandarlaki N., Darvishmohammadi T. (2013). Nanolipodendrosome-loaded glatiramer acetate and myogenic differentiation 1 as augmentation therapeutic strategy approaches in muscular dystrophy. Int. J. Nanomed..

[180] Turjeman K., Yanay N., Elbaz M., Bavli Y., Gross M., Rabie M., Barenholz Y., Nevo Y. (2019). Liposomal steroid nano-drug is superior to steroids as-is in *mdx* mouse model of Duchenne muscular mystrophy. Nanomedicine.

[181] Yukihara M., Ito K., Tanoue O., Goto K., Matsushita T., Matsumoto Y., Masuda M., Kimura S., Ueoka R. (2011). Effective drug delivery system for Duchenne muscular dystrophy using hybrid liposomes including gentamicin along with reduced toxicity. Biol. Pharm. Bull..

[182] Bibee K.P., Cheng Y., Ching J.K., Marsh J.N., Li A.J., Keeling R.M., Connolly A.M., Golumbek P.T., Myerson J.W., Hu G. (2014). Rapamycin nanoparticles target defective autophagy in muscular dystrophy to enhance both strength and cardiac function. FASEB J..

[183] Wei T., Cheng Q., Min Y.-L., Olson E.N., Siegwart D.J. (2020). Systemic nanoparticle delivery of CRISPR-Cas9 ribonucleoproteins for effective tissue specific genome editing. Nat. Commun..

[184] Lee K., Conboy M., Park H.M., Jiang F., Kim H.J., Dewitt M.A., Mackley V.A., Chang K., Rao A., Skinner C. (2017). Nanoparticle delivery of Cas9 ribonucleoprotein and donor DNA in vivo induces homology-directed DNA repair. Nat. Biomed. Eng..

[185] Järver P., O’Donovan L., Gait M.J. (2014). A chemical view of oligonucleotides for exon skipping and related drug applications. Nucleic Acid Ther..

[186] Zhang P., Wagner E. (2017). History of polymeric gene delivery systems. Top. Curr. Chem..

[187] Boussif O., Lezoualc’h F., Zanta M.A., Mergny M.D., Scherman D., Demeneix B., Behr J.P. (1995). A versatile vector for gene and oligonucleotide transfer into cells in culture and *in vivo*: Polyethylenimine. Proc. Natl. Acad. Sci. USA.

[188] Lu Q.L., Mann C.J., Lou F., Bou-Gharios G., Morris G.E., Xue S., Fletcher S., Partridge T.A., Wilton S.D. (2003). Functional amounts of dystrophin produced by skipping the mutated exon in the *mdx* dystrophic mouse. Nat. Med..

[189] Lu Q.L., Bou-Gharios G., Partridge T.A. (2003). Non-viral gene delivery in skeletal muscle: A protein factory. Gene Ther..

[190] Williams J.H., Sirsi S.R., Latta D.R., Lutz G.J. (2006). Induction of dystrophin expression by exon skipping in *mdx* mice following intramuscular injection of antisense oligonucleotides complexed with PEG–PEI copolymers. Mol. Ther..

[191] Lutz G.J., Sirsi S.R., Williams J.H. (2008). PEG-PEI copolymers for oligonucleotide delivery to Cells and tissues. Methods Mol. Biol..

[192] Castaldello A., Brocca-Cofano E., Voltan R., Triulzi C., Altavilla G., Laus M., Sparnacci K., Ballestri M., Tondelli L., Fortini C. (2006). DNA prime and protein boost immunization with innovative polymeric cationic core-shell nanoparticles elicits broad immune responses and strongly enhance cellular responses of HIV-1 tat DNA vaccination. Vaccine.

[193] Summerton J., Weller D. (1997). Morpholino antisense oligomers: Design, preparation, and properties. Antisense Nucleic Acid Drug Dev..

[194] Miller C.M., Harris E.N. (2016). Antisense oligonucleotides: Treatment strategies and cellular internalization. RNA Dis..

[195] Järver P., Zaghloul E.M., Arzumanov A.A., Saleh A.F., McClorey G., Hammond S.M., Hällbrink M., Langel Ü., Smith C.I.E., Wood M.J.A. (2015). Peptide nanoparticle delivery of charge-neutral splice-switching morpholino oligonucleotides. Nucleic Acid Ther..

[196] Suzuki R., Takizawa T., Negishi Y., Hagisawa K., Tanaka K., Sawamura K., Utoguchi N., Nishioka T., Maruyama K. (2007). Gene delivery by combination of novel liposomal bubbles with perfluoropropane and ultrasound. J. Control. Release.

[197] Negishi Y., Endo Y., Fukuyama T., Suzuki R., Takizawa T., Omata D., Maruyama K., Aramaki Y. (2008). Delivery of siRNA into the cytoplasm by liposomal bubbles and ultrasound. J. Control. Release.

[198] Tros de Ilarduya C., Sun Y., Düzgüneş N. (2010). Gene delivery by lipoplexes and polyplexes. Eur. J. Pharm. Sci..

[199] Kulkarni J.A., Cullis P.R., van der Meel R. (2018). Lipid nanoparticles enabling gene therapies: From concepts to clinical utility. Nucleic Acid Ther..

[200] Li D., Sharili A.S., Connelly J., Gautrot J.E. (2018). Highly stable RNA capture by dense cationic polymer brushes for the design of cytocompatible, serum-stable siRNA delivery vectors. Biomacromolecules.

[201] Kulkarni J.A., Myhre J.L., Chen S., Tam Y.Y.C., Danescu A., Richman J.M., Cullis P.R. (2017). Design of lipid nanoparticles for in vitro and in vivo delivery of plasmid DNA. Nanomedicine.

[202] Sano A., Maeda M., Nagahara S., Ochiya T., Honma K., Itoh H., Miyata T., Fujioka K. (2003). Atelocollagen for protein and gene delivery. Adv. Drug Deliv. Rev..

[203] Ochiya T., Nagahara S., Sano A., Itoh H., Terada M. (2001). Biomaterials for gene delivery: Atelocollagen-mediated controlled release of molecular medicines. Curr. Gene Ther..

[204] Wang J., Mao H.-Q., Leong K.W. (2001). A novel biodegradable gene carrier based on polyphosphoester. J. Am. Chem. Soc..

[205] Itaka K., Yamauchi K., Harada A., Nakamura K., Kawaguchi H., Kataoka K. (2003). Polyion complex micelles from plasmid DNA and poly(ethylene glycol)-poly(L-lysine) block copolymer as serum-tolerable polyplex system: Physicochemical properties of micelles relevant to gene transfection efficiency. Biomaterials.

[206] Parvathaneni V., Kulkarni N.S., Muth A., Gupta V. (2019). Drug repurposing: A promising tool to accelerate the drug discovery process. Drug Discov. Today.

[207] He H., Markoutsa E., Li J., Xu P. (2018). Repurposing disulfiram for cancer therapy via targeted nanotechnology through enhanced tumor mass penetration and disassembly. Acta Biomater..

[208] Amini M.A., Abbasi A.Z., Cai P., Lip H., Gordijo C.R., Li J., Chen B., Zhang L., Rauth A.M., Wu X.Y. (2019). Combining tumor microenvironment modulating nanoparticles with doxorubicin to enhance chemotherapeutic efficacy and boost antitumor immunity. J. Natl. Cancer Inst..

[209] Lim S., Park J., Shim M.K., Um W., Yoon H.Y., Ryu J.H., Lim D.-K., Kim K. (2019). Recent advances and challenges of repurposing nanoparticle-based drug delivery systems to enhance cancer immunotherapy. Theranostics.

[210] Gregoriou Y., Gregoriou G., Yilmaz V., Kapnisis K., Prokopi M., Anayiotos A., Strati K., Dietis N., Constantinou A.I., Andreou C. (2021). Resveratrol loaded polymeric micelles for theranostic targeting of breast cancer cells. Nanotheranostics.

[211] Dunant P., Walter M.C., Karpati G., Lochmüller H. (2003). Gentamicin fails to increase dystrophin expression in dystrophin-deficient muscle. Muscle Nerve.

[212] Zammit P.S. (2017). Function of the myogenic regulatory factors Myf5, MyoD, Myogenin and MRF4 in skeletal muscle, satellite cells and regenerative myogenesis. Semin. Cell Dev. Biol..

[213] Sehgal S.N. (2003). Sirolimus: Its discovery, biological properties, and mechanism of action. Transplant. Proc..

[214] Flaim S.F. (1994). Pharmacokinetics and side effects of perfluorocarbon-based blood substitutes. Artif. Cells. Blood Substit. Immobil. Biotechnol..

[215] Spahn D.R. (1999). Blood substitutes. Artificial oxygen carriers: Perfluorocarbon emulsions. Crit. Care.

[216] Watanabe A., Tanaka H., Sakurai Y., Tange K., Nakai Y., Ohkawara T., Takeda H., Harashima H., Akita H. (2016). Effect of particle size on their accumulation in an inflammatory lesion in a dextran sulfate sodium (DSS)-induced colitis model. Int. J. Pharm..

[217] Chen K.-H., Lundy D.J., Toh E.K.-W., Chen C.-H., Shih C., Chen P., Chang H.-C., Lai J.J., Stayton P.S., Hoffman A.S. (2015). Nanoparticle distribution during systemic inflammation is size-dependent and organ-specific. Nanoscale.

[218] Jinek M., Chylinski K., Fonfara I., Hauer M., Doudna J.A., Charpentier E. (2012). A programmable dual-RNA-guided DNA endonuclease in adaptive bacterial immunity. Science.

[219] Luther D.C., Lee Y.W., Nagaraj H., Scaletti F., Rotello V.M. (2018). Delivery approaches for CRISPR/Cas9 therapeutics *in vivo*: Advances and challenges. Expert Opin. Drug Deliv..

[220] Lino C.A., Harper J.C., Carney J.P., Timlin J.A. (2018). Delivering CRISPR: A review of the challenges and approaches. Drug Deliv..

[221] Ding Y., Jiang Z., Saha K., Kim C.S., Kim S.T., Landis R.F., Rotello V.M. (2014). Gold nanoparticles for nucleic acid delivery. Mol. Ther..

[222] Chithrani B.D., Ghazani A.A., Chan W.C.W. (2006). Determining the size and shape dependence of gold nanoparticle uptake into mammalian cells. Nano Lett..

[223] Arandel L., Polay Espinoza M., Matloka M., Bazinet A., De Dea Diniz D., Naouar N., Rau F., Jollet A., Edom-Vovard F., Mamchaoui K. (2017). Immortalized human myotonic dystrophy muscle cell lines to assess therapeutic compounds. Dis. Model. Mech..

[224] Matloka M., Klein A.F., Rau F., Furling D. (2018). Cells of matter—*in vitro* models for myotonic dystrophy. Front. Neurol..

[225] Bigot A., Klein A.F., Gasnier E., Jacquemin V., Ravassard P., Butler-Browne G., Mouly V., Furling D. (2009). Large CTG repeats trigger P16-dependent premature senescence in myotonic dystrophy type 1 muscle precursor cells. Am. J. Pathol..

[226] Hayflick L. (1965). The limited in vitro lifetime of human diploid cell strains. Exp. Cell Res..

[227] Renault V., Piron-Hamelin G., Forestier C., DiDonna S., Decary S., Hentati F., Saillant G., Butler-Browne G.S., Mouly V. (2000). Skeletal muscle regeneration and the mitotic clock. Exp. Gerontol..

[228] Renna L.V., Cardani R., Botta A., Rossi G., Fossati B., Costa E., Meola G. (2014). Premature senescence in primary muscle cultures of myotonic dystrophy type 2 is not associated with P16 induction. Eur. J. Histochem..

[229] Thornell L.-E., Lindstöm M., Renault V., Klein A., Mouly V., Ansved T., Butler-Browne G., Furling D. (2009). Satellite cell dysfunction contributes to the progressive muscle atrophy in myotonic dystrophy type 1. Neuropathol. Appl. Neurobiol..

[230] Liang R., Dong W., Shen X., Peng X., Aceves A.G., Liu Y. (2016). Modeling myotonic dystrophy 1 in C2C12 myoblast cells. J. Vis. Exp..

[231] Mamchaoui K., Trollet C., Bigot A., Negroni E., Chaouch S., Wolff A., Kandalla P.K., Marie S., Di Santo J., St Guily J.L. (2011). Immortalized pathological human myoblasts: Towards a universal tool for the study of neuromuscular disorders. Skelet. Muscle.

[232] O’Connor M.S., Carlson M.E., Conboy I.M. (2009). Differentiation rather than aging of muscle stem cells abolishes their telomerase activity. Biotechnol. Prog..

[233] Massenet J., Gitiaux C., Magnan M., Cuvellier S., Hubas A., Nusbaum P., Dilworth F.J., Desguerre I., Chazaud B. (2020). Derivation and characterization of immortalized human muscle satellite cell clones from muscular dystrophy patients and healthy individuals. Cells.

[234] Chaouch S., Mouly V., Goyenvalle A., Vulin A., Mamchaoui K., Negroni E., Di Santo J., Butler-Browne G., Torrente Y., Garcia L. (2009). Immortalized skin fibroblasts expressing conditional MyoD as a renewable and reliable source of converted human muscle cells to assess therapeutic strategies for muscular mystrophies: Validation of an exon-skipping approach to restore dystrophin in Duchenne muscular dystrophy Cells. Hum. Gene Ther..

[235] Cooper S.T., Kizana E., Yates J.D., Lo H.P., Yang N., Wu Z.H., Alexander I.E., North K.N. (2007). Dystrophinopathy carrier determination and detection of protein deficiencies in muscular dystrophy using lentiviral MyoD-forced myogenesis. Neuromuscul. Disord..

[236] Edmondson R., Broglie J.J., Adcock A.F., Yang L. (2014). Three-dimensional cell culture systems and their applications in drug discovery and cell-based biosensors. Assay Drug Dev. Technol..

[237] Duval K., Grover H., Han L.-H., Mou Y., Pegoraro A.F., Fredberg J., Chen Z. (2017). Modeling physiological events in 2D vs. 3D cell culture. Physiology.

[238] Knight E., Przyborski S. (2015). Advances in 3D cell culture technologies enabling tissue-like structures to be created *in vitro*. J. Anat..

[239] Jeffries G.D.M., Xu S., Lobovkina T., Kirejev V., Tusseau F., Gyllensten C., Singh A.K., Karila P., Moll L., Orwar O. (2020). 3D micro-organisation printing of mammalian cells to generate biological tissues. Sci. Rep..

[240] Liu Y., Wu B., Gong L., An C., Lin J., Li Q., Jiang D., Jin K., Mechakra A., Bunpetch V. (2019). Dissecting cell diversity and connectivity in skeletal muscle for myogenesis. Cell Death Dis..

[241] Juhas M., Engelmayr G.C., Fontanella A.N., Palmer G.M., Bursac N. (2014). Biomimetic Engineered muscle with capacity for vascular integration and functional maturation *in vivo*. Proc. Natl. Acad. Sci. USA.

[242] Borselli C., Cezar C.A., Shvartsman D., Vandenburgh H.H., Mooney D.J. (2011). The role of multifunctional delivery scaffold in the ability of cultured myoblasts to promote muscle regeneration. Biomaterials.

[243] McLean I.C., Schwerdtfeger L.A., Tobet S.A., Henry C.S. (2018). Powering ex vivo tissue models in microfluidic systems. Lab. Chip.

[244] Gowing G., Svendsen S., Svendsen C.N. (2017). Ex vivo gene therapy for the treatment of neurological disorders. Prog. Brain Res..

[245] Mobini S., Song Y.H., McCrary M.W., Schmidt C.E. (2019). Advances in ex vivo models and lab-on-a-chip devices for neural tissue engineering. Biomaterials.

[246] Smith L.R., Meyer G.A. (2020). Skeletal muscle explants: *Ex-vivo* models to study cellular behavior in a complex tissue environment. Connect. Tissue Res..

[247] Kim D., Wu X., Young A.T., Haynes C.L. (2014). Microfluidics-based in vivo mimetic systems for the study of cellular biology. Acc. Chem. Res..

[248] Carton F., Calderan L., Malatesta M. (2017). Incubation under fluid dynamic conditions markedly improves the structural preservation in vitro of explanted skeletal muscles. Eur. J. Histochem..

[249] Carosio S., Barberi L., Rizzuto E., Nicoletti C., Del Prete Z., Musarò A. (2013). Generation of *ex vivo*-vascularized muscle engineered tissue (X-MET). Sci. Rep..

[250] Fischer H.C., Chan W.C. (2007). Nanotoxicity: The growing need for in vivo study. Curr. Opin. Biotechnol..

[251] Bostrom M., O’Keefe R. (2008). What experimental approaches (eg, *in vivo*, *in vitro*, tissue retrieval) are effective in investigating the biologic effects of particles?. J. Am. Acad. Orthop. Surg..

[252] McGreevy J.W., Hakim C.H., McIntosh M.A., Duan D. (2015). Animal models of Duchenne muscular dystrophy: From basic mechanisms to gene therapy. Dis. Model. Mech..

[253] Wells D.J. (2018). Tracking progress: An update on animal models for Duchenne muscular dystrophy. Dis. Model. Mech..

[254] Collins C.A., Morgan J.E. (2003). Duchenne’s muscular dystrophy: Animal models used to investigate pathogenesis and develop therapeutic strategies. Int. J. Exp. Pathol..

[255] Bulfield G., Siller W.G., Wight P.A., Moore K.J. (1984). X Chromosome-linked muscular dystrophy (*mdx*) in the mouse. Proc. Natl. Acad. Sci. USA.

[256] van Putten M., Putker K., Overzier M., Adamzek W.A., Pasteuning-Vuhman S., Plomp J.J., Aartsma-Rus A. (2019). Natural disease history of the D2-*mdx* mouse model for Duchenne muscular dystrophy. FASEB J..

[257] Desguerre I., Arnold L., Vignaud A., Cuvellier S., Yacoub-Youssef H., Gherardi R.K., Chelly J., Chretien F., Mounier R., Ferry A. (2012). A new model of experimental fibrosis in hindlimb skeletal muscle of adult *mdx* mouse mimicking muscular dystrophy. Muscle Nerve.

[258] Ng R., Banks G.B., Hall J.K., Muir L.A., Ramos J.N., Wicki J., Odom G.L., Konieczny P., Seto J., Chamberlain J.R. (2012). Animal models of muscular dystrophy. Prog. Mol. Biol. Transl. Sci..

[259] Kornegay J.N. (2017). The golden retriever model of Duchenne muscular dystrophy. Skelet. Muscle.

[260] Kawahara G., Karpf J.A., Myers J.A., Alexander M.S., Guyon J.R., Kunkel L.M. (2011). Drug screening in a zebrafish model of Duchenne muscular dystrophy. Proc. Natl. Acad. Sci. USA.

[261] Widrick J.J., Kawahara G., Alexander M.S., Beggs A.H., Kunkel L.M. (2019). Discovery of novel therapeutics for muscular dystrophies using zebrafish phenotypic screens. J. Neuromuscul. Dis..

[262] Sicot G., Gomes-Pereira M. (2013). RNA toxicity in human disease and animal models: From the uncovering of a new mechanism to the development of promising therapies. Biochim. Biophys. Acta.

[263] Gomes-Pereira M., Cooper T.A., Gourdon G. (2011). Myotonic dystrophy mouse models: Towards rational therapy development. Trends Mol. Med..

[264] Guiraud-Dogan C., Huguet A., Gomes-Pereira M., Brisson E., Bassez G., Junien C., Gourdon G. (2007). DM1 CTG expansions affect insulin receptor isoforms expression in various tissues of transgenic mice. Biochim. Biophys. Acta.

[265] Lin X., Miller J.W., Mankodi A., Kanadia R.N., Yuan Y., Moxley R.T., Swanson M.S., Thornton C.A. (2006). Failure of MBNL1-dependent post-natal splicing transitions in myotonic dystrophy. Hum. Mol. Genet..

[266] Mankodi A., Logigian E., Callahan L., McClain C., White R., Henderson D., Krym M., Thornton C.A. (2000). Myotonic dystrophy in transgenic mice expressing an expanded CUG repeat. Science.

[267] Mankodi A., Takahashi M.P., Jiang H., Beck C.L., Bowers W.J., Moxley R.T., Cannon S.C., Thornton C.A. (2002). Expanded CUG repeats trigger aberrant splicing of ClC-1 chloride channel pre-mRNA and hyperexcitability of skeletal muscle in myotonic dystrophy. Mol. Cell.

[268] Seznec H., Agbulut O., Sergeant N., Savouret C., Ghestem A., Tabti N., Willer J.C., Ourth L., Duros C., Brisson E. (2001). Mice transgenic for the human myotonic dystrophy region with expanded CTG repeats display muscular and brain abnormalities. Hum. Mol. Genet..

[269] Hinman M.N., Richardson J.I., Sockol R.A., Aronson E.D., Stednitz S.J., Murray K.N., Berglund J.A., Guillemin K. (2020). Zebrafish mbnl mutants model physical and molecular phenotypes of myotonic dystrophy. bioRxiv.

[270] Machuca-Tzili L.E., Buxton S., Thorpe A., Timson C.M., Wigmore P., Luther P.K., Brook J.D. (2011). Zebrafish deficient for Muscleblind-like 2 exhibit features of myotonic dystrophy. Dis. Model. Mech..

[271] Carton F., Chevalier Y., Nicoletti L., Tarnowska M., Stella B., Arpicco S., Malatesta M., Jordheim L.P., Briançon S., Lollo G. (2019). Rationally designed hyaluronic acid-based nano-complexes for pentamidine delivery. Int. J. Pharm..

[272] Stella B., Andreana I., Zonari D., Arpicco S. (2020). Pentamidine-loaded lipid and polymer nanocarriers as tunable anticancer drug delivery systems. J. Pharm. Sci..

[273] Falzarano M.S., Bassi E., Passarelli C., Braghetta P., Ferlini A. (2014). Biodistribution studies of polymeric nanoparticles for drug delivery in mice. Hum Gene Ther..

[274] Huang D., Yue F., Qiu J., Deng M., Kuang S. (2020). Polymeric nanoparticles functionalized with muscle-homing peptides for targeted delivery of phosphatase and tensin homolog inhibitor to skeletal muscle. Acta Biomater..

[275] Herweijer H., Wolff J.A. (2007). Gene therapy progress and prospects: Hydrodynamic gene delivery. Gene Ther..

[276] Kumbhari V., Li L., Piontek K., Ishida M., Fu R., Khalil B., Garrett C.M., Liapi E., Kalloo A.N., Selaru F.M. (2018). Successful liver-directed gene delivery by ERCP-guided hydrodynamic injection (with Videos). Gastrointest. Endosc..

[277] Le Guen Y.T., Le Gall T., Midoux P., Guégan P., Braun S., Montier T. (2020). Gene transfer to skeletal muscle using hydrodynamic limb vein injection: Current applications, hurdles and possible optimizations. J. Gene Med..

[278] Ashfaq U.A., Riaz M., Yasmeen E., Yousaf M. (2017). Recent advances in nanoparticle-based targeted drug-delivery systems against cancer and role of tumor microenvironment. Crit. Rev. Ther. Drug Carrier Syst..

[279] Pietersz G.A., Wang X., Yap M.L., Lim B., Peter K. (2017). Therapeutic targeting in nanomedicine: The future lies in recombinant antibodies. Nanomedicine.

[280] Arahata K., Ishiura S., Ishiguro T., Tsukahara T., Suhara Y., Eguchi C., Ishiharat T., Nonaka I., Ozawa E., Sugita H. (1988). Immunostaining of skeletal and cardiac muscle surface membrane with antibody against Duchenne muscular dystrophy peptide. Nature.

[281] Zhang R., Kim A.S., Fox J.M., Nair S., Basore K., Klimstra W.B., Rimkunas R., Fong R.H., Lin H., Poddar S. (2018). Mxra8 is a receptor for multiple arthritogenic alphaviruses. Nature.

[282] Poon W., Zhang X., Bekah D., Teodoro J.G., Nadeau J.L. (2015). Targeting B16 tumors in vivo with peptide-conjugated gold nanoparticles. Nanotechnology.

[283] Yu-Wai-Man C., Tagalakis A.D., Manunta M.D., Hart S.L., Khaw P.T. (2016). Receptor-targeted liposome-peptide-siRNA nanoparticles represent an efficient delivery system for MRTF silencing in conjunctival fibrosis. Sci. Rep..

[284] Tajau R., Rohani R., Abdul Hamid S.S., Adam Z., Mohd Janib S.N., Salleh M.Z. (2020). Surface functionalisation of poly-APO- b -polyol ester cross-linked copolymers as core–shell nanoparticles for targeted breast cancer therapy. Sci. Rep..

[285] Yu C.-Y., Yuan Z., Cao Z., Wang B., Qiao C., Li J., Xiao X. (2009). A muscle-targeting peptide displayed on AAV2 improves muscle tropism on systemic delivery. Gene Ther..

[286] Seow Y., Yin H., Wood M.J.A. (2010). Identification of a novel muscle targeting peptide in *mdx* mice. Peptides.

[287] Tsoumpra M.K., Fukumoto S., Matsumoto T., Takeda S., Wood M.J.A., Aoki Y. (2019). Peptide-conjugate antisense based splice-correction for Duchenne muscular dystrophy and other neuromuscular diseases. EBioMedicine.

[288] Gao X., Zhao J., Han G., Zhang Y., Dong X., Cao L., Wang Q., Moulton H.M., Yin H. (2014). Effective dystrophin restoration by a novel muscle-homing peptide-morpholino conjugate in dystrophin-deficient *mdx* mice. Mol. Ther..

